# Altered membrane rigidity via enhanced endogenous cholesterol synthesis drives cancer cell resistance to destruxins

**DOI:** 10.18632/oncotarget.25432

**Published:** 2018-05-22

**Authors:** Daniela Heilos, Clemens Röhrl, Christine Pirker, Bernhard Englinger, Dina Baier, Thomas Mohr, Michaela Schwaiger, Shahid Muhammad Iqbal, Sushilla van Schoonhoven, Kristaps Klavins, Tanja Eberhart, Ursula Windberger, Judith Taibon, Sonja Sturm, Hermann Stuppner, Gunda Koellensperger, Rita Dornetshuber-Fleiss, Walter Jäger, Rosa Lemmens-Gruber, Walter Berger

**Affiliations:** ^1^ Institute of Cancer Research, Department of Internal Medicine I, Medical University of Vienna, Comprehensive Cancer Center of the Medical University of Vienna, Vienna, Austria; ^2^ Department of Pharmacology and Toxicology, University of Vienna, Vienna, Austria; ^3^ Center for Pathobiochemistry and Genetics, Medical University of Vienna, Vienna, Austria; ^4^ Decentralized Biomedical Facilities of the Medical University of Vienna, Vienna, Austria; ^5^ Department of Analytical Chemistry, Faculty of Chemistry, University of Vienna, Vienna, Austria; ^6^ BIOCRATES Life Sciences AG, Innsbruck, Austria; ^7^ Institute of Pharmacy, Pharmacognosy and Center for Molecular Biosciences Innsbruck, University of Innsbruck, Innsbruck, Austria; ^8^ Vienna Metabolomics Center, University of Vienna, Vienna, Austria; ^9^ Department of Pharmaceutical Chemistry, Division of Clinical Pharmacy and Diagnostics, University of Vienna, Vienna, Austria

**Keywords:** cholesterol synthesis pathway, cancer cell resistance, destruxins, mycotoxins, cell membrane alterations

## Abstract

Destruxins, secondary metabolites of entomopathogenic fungi, exert a wide variety of interesting characteristics ranging from antiviral to anticancer effects. Although their mode of action was evaluated previously, the molecular mechanisms of resistance development are unknown. Hence, we have established destruxin-resistant sublines of HCT116 colon carcinoma cells by selection with the most prevalent derivatives, destruxin (dtx)A, dtxB and dtxE. Various cell biological and molecular techniques were applied to elucidate the regulatory mechanisms underlying these acquired and highly stable destruxin resistance phenotypes. Interestingly, well-known chemoresistance-mediating ABC efflux transporters were not the major players. Instead, in dtxA- and dtxB-resistant cells a hyper-activated mevalonate pathway was uncovered resulting in increased *de-novo* cholesterol synthesis rates and elevated levels of lanosterol, cholesterol as well as several oxysterol metabolites. Accordingly, inhibition of the mevalonate pathway at two different steps, using either statins or zoledronic acid, significantly reduced acquired but also intrinsic destruxin resistance. Vice versa, cholesterol supplementation protected destruxin-sensitive cells against their cytotoxic activity. Additionally, an increased cell membrane adhesiveness of dtxA-resistant as compared to parental cells was detected by atomic force microscopy. This was paralleled by a dramatically reduced ionophoric capacity of dtxA in resistant cells when cultured in absence but not in presence of statins. Summarizing, our results suggest a reduced ionophoric activity of destruxins due to cholesterol-mediated plasma membrane re-organization as molecular mechanism underlying acquired destruxin resistance in human colon cancer cells. Whether this mechanism might be valid also in other cell types and organisms exposed to destruxins e.g. as bio-insecticides needs to be evaluated.

## INTRODUCTION

According to the World Health Organization (WHO), 8.8 million people died from cancer in the year 2015, making malignant diseases the second leading cause of death worldwide [1–3]. As cancer is a multifactorial disease a vast variety of different anticancer agents have been developed in the last decades [4] for systemic administration, such as chemo- or immunotherapies. Nonetheless, severe adverse reactions, due to primary unresponsiveness of the tumor to systemic therapies or the treatment-induced secondary development of multidrug resistance resulting in tumor relapse, both progression-free and overall survival of many cancer patients are still unsatisfying [5–7].

Hence, the search for novel anticancer drugs is still ongoing. One focus is set on natural products including peptidic secondary metabolites isolated from diverse organisms such as fungi, microorganisms and plants [8, 9]. For instance, the depsipeptide romidepsin (FK-228, Istodax^®^), isolated from *Chromobacterium violaceum,* was approved for the treatment of relapsing or refractory T-cell lymphoma in 2009 [10]. Additionally, the structurally related cyclic depsipeptides enniatin and beauvericin are fungal metabolites with promising anticancer effects *in vitro* [11–14] and *in vivo* [15, 16].

Another interesting group of cyclic depsipeptides are destruxins first isolated in 1961 from the entomopathogenic fungus *Metarhizium brunneum* [17]. The three most prevalent isoforms are destruxin A (dtxA), destruxin B (dtxB) and destruxin E (dtxE) [18]. Destruxins exhibit a great variety of biological activities ranging from insecticidal, phytotoxic and antiviral effects to antiangiogenic, antiproliferative and cytotoxic properties in cancer cells [19, 20].

Accordingly, destruxins are discussed as candidates for the development of novel therapeutics for the treatment of diverse maladies such as hepatitis B [21–24], liver fibrosis [25], osteoporosis [26] or Alzheimer’s disease [27]. In the field of cancer research, destruxins have been investigated *in vitro* for their therapeutic potential against oral carcinomas [28], leukemia [29–31], lymphomas [32], non-small cell lung cancer [33], hepatocellular carcinoma especially in combination with the tyrosine kinase inhibitor sorafenib [34], and colorectal cancer [20]. Additionally, significant anticancer activity of dtxB was reported *in vivo* against colorectal cancer in two studies using HT-29 xenograft mouse models without observing any dtxB-related adverse effects [35, 36].

The mode of action of destruxins was found to be multifaceted, probably based on their calcium ion interactions and ionophoric properties [37]. Additionally, the activation of the intrinsic mitochondrial apoptotic pathway [20, 34] as well as apoptosis induction via the death receptor pathway, i.e. the Fas associated death domain (FADD), was shown [32]. In some studies, a cell cycle arrest (G0/G1 or S phase), depending on the cell line investigated, was also observed after administraion of destruxins [20, 30]. The treatment of cancer cells with dtxE resulted in growth inhibition which was mediated by a decrease in cyclin D1 levels [20, 38]. Furthermore, blockade of the Wnt/β-catenin [28, 35] and the phosphoinositide-3-kinase (PI3K)/Akt signaling pathways [20, 35] was discussed to be involved in the cytotoxic activity of destruxins. One study [26] suggested that the anticancer activity of destruxins was based on their inhibitory effects on the vacuolar-type H^+^-ATPase (V-ATPase) [39, 40].

However, to further develop the therapeutic potential of destruxins, besides their anticancer activity and toxicological characteristics, acquired resistance mechanisms, which might arise during long-term therapy, need to be investigated in greater detail. As previous *in vivo* reports have suggested activity of dtxB against colorectal cancer [35, 36], the present study focused on the establishment of colorectal carcinoma cell models with acquired destruxin resistance based on long-term drug selection. This approach enabled us 1) to identify the molecular mechanisms of acquired destruxin-resistance and 2) to propose strategies to re-establish destruxin sensitivity after resistance to destruxin-treatment had occurred.

## RESULTS

### Selection against increasing dtx concentrations resulted in sublines with stable resistance to dtxA, dtxB or dtxE

**Figure 1:**
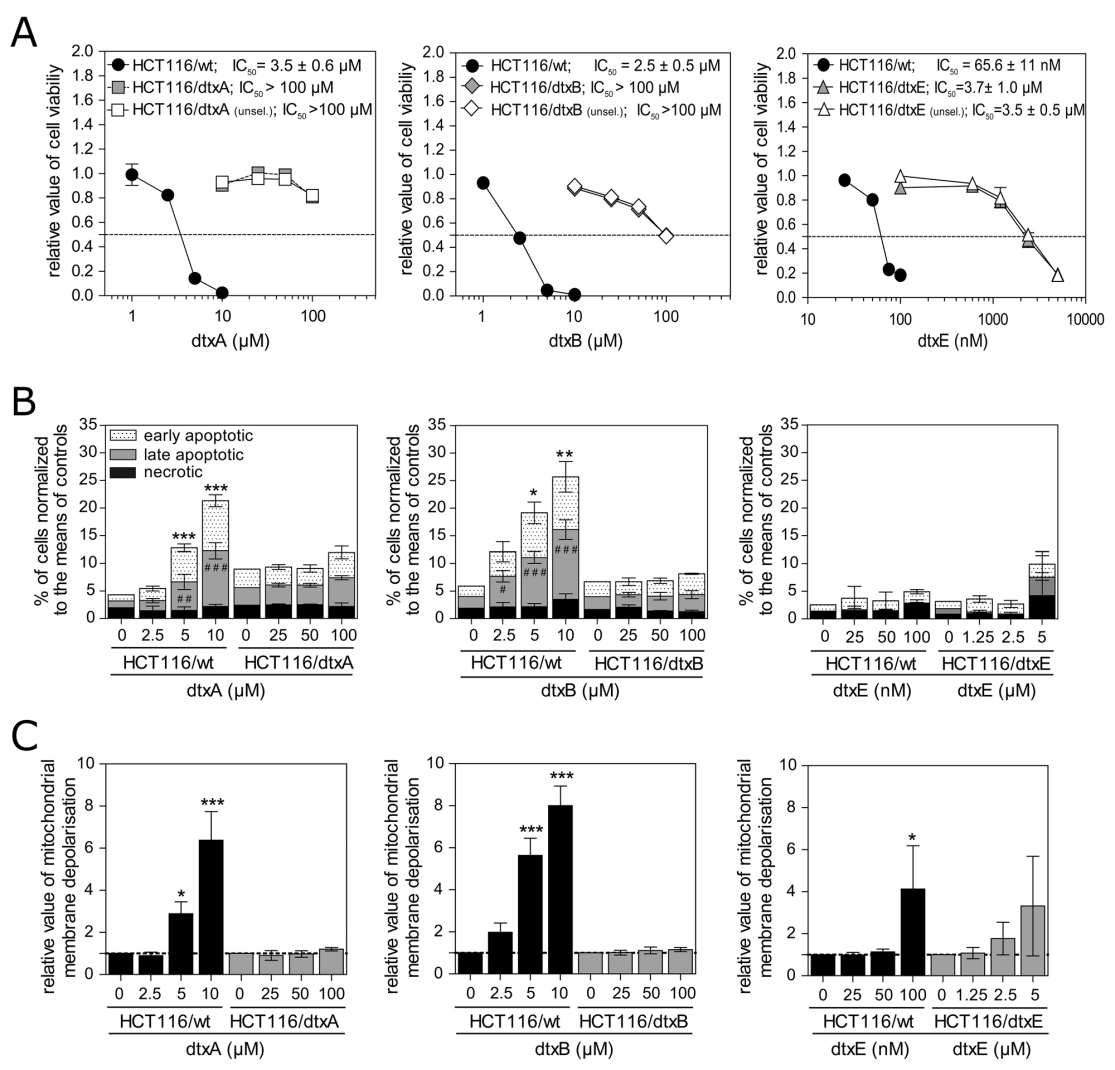
DtxA-, dtxB- and dtxE-selected sublines tolerate high concentrations of the corresponding dtx **(A)** Cell viability of parental cells compared to dtxA- (left), dtxB- (middle) and dtxE-resistant (right) sublines after 72 h treatment with the indicated concentrations of dtxA, dtxB and dtxE, respectively, is shown. Open symbols indicate sublines resistant to dtxA, dtxB and dtxE that had been removed from drug selection (unsel) for one month prior to this experiment. Results from one representative out of at least three independent experiment are displayed. Values are given as mean (± SD). Dashed lines indicate 50% cell viability and respective mean IC_50_-values (n ≥ 3) are displayed in the upper part of the panels. **(B)** Mean percentages (± SD) of early and late apoptotic and of necrotic cells were determined by Annexin-V/PI staining in three independent experiments after treatment of parental and resistant sublines with the indicated concentrations of dtxA (left), dtxB (middle) and dtxE (right) for 48 h. Hashtags (#) and asterisks (^*^) indicate significant differences between late and early apoptotic rates, respectively, compared to untreated controls. **(C)** Mitochondrial membrane depolarization of parental cells, compared to the respective dtxA- (left), dtxB- (middle) and dtxE-resistant sublines (right) that had been treated with the indicated concentrations of dtxA (right), dtxB (middle) or dtxE (left) for 48 h. Mean values (± SD) of three independent experiments are shown relative to untreated controls set as 1. Significant differences to untreated controls are marked by asterisks (^*^p<0.05; ^**^p<0.01; ^***^p<0.001).

As published previously, dtxA and dtxB at concentrations in the low micromolar range and in case of dtxE even the nanomolar range significantly reduced cell viability of multiple cancer cell lines including HCT116 colon cancer cells [20] with IC_50_ values of 3.5 ± 0.6 μM, 2.5 ± 0.5 μM and 65.6 ± 11 nM for dtxA, dtxB and dtxE, respectively. For induction of resistance, HCT116/wt cells were grown with increasing concentrations of dtxA, dtxB or dtxE and a rapid development of resistance was observed. After four months, a 1.3, 4.4 and 3.8-fold tolerance was determined for dtxA, dtxB and dtxE, respectively, in the particular sublines increasing gradually during the complete period of observation (Supplementary Table 5). After approximately one year, IC_50_ values were higher than 100 μM for both HCT116/dtxA and HCT116/dtxB with a >28.6 and >40-fold resistance, respectively, compared to the parental HCT116/wt cells (Figure 1A, gray symbols; Table 1; Supplementary Table 5). For the HCT116/dtxE cells a 55.7-fold resistance was obtained with a mean IC_50_ value of 3.7 μM (Figure 1A, right panel; Table 1; Supplementary Table 5).

**Table 1:** Resistance/cross-resistance of HCT116/wt cells and the three destruxin-selected sublines to dtxA, dtxB and dtxE

Cell line	dtxA		dtxB		dtxE	
	IC_50_^#^ (μM)	relative resistance^§^ (-fold)	IC_50_^#^ (μM)	relative resistance^§^ (-fold)	IC_50_^#^ (nM)	relative resistance^§^ (-fold)
HCT116/wt	3.5 ± 0.6		2.5 ± 0.5		65 ± 11	
HCT116/dtxA	>100	>28.6	45 ± 29	18.2	>5000	>76.2
HCT116/dtxB	76 ± 34	21.8	>100	>40	3854 ± 1621	58.8
HCT116/dtxE	1.0 ± 0.2	0.3	0.8 ± 0.2	0.3	3653 ± 1060	55.7

^#^ IC_50_ values were calculated from dose-response curves and are given as means (± SD) from at least three independent experiments performed in triplicates.

^§^ Relative resistance was calculated by dividing the mean IC_50_ value of the respective resistant cell line by the IC_50_ value of the parental cell line HCT116/wt.

Resistance of sublines to the respective destruxins remained unchanged after cells were removed from selection pressure for one month. Cell viability assays revealed almost identical cell growth curves compared to resistant sublines continuously grown under the selection pressure of the distinctive destruxin (Figure 1A, open symbols).

Previously, apoptosis induction involving mitochondrial membrane depolarization, indicative for intrinsic cell death pathway activation, has been reported as mode of action of destruxins in colorectal carcinoma cells [20]. We found that the percentages of early and/or late apoptotic cells were already markedly and significantly increased in the parental cell line treated with 5 μM of dtxA or with 2.5 μM of dtxB, at concentrations comparable to the IC_50_ values obtained in the MTT assays (Figure 1B). In contrast, up to 100 μM of the respective destruxin derivative, representing a 20- to 40-fold increase of the concentration already active in the parental cell line, did not induce a relevant increase of early and late apoptosis or of necrosis rates in the resistant sublines (Figure 1B, left and middle panel).

Concerning dtxE, at concentrations around the IC_50_ value only a minor increase in the percentage of cells in early and late apoptosis or necrosis was observed in parental HCT116 cells, indicating that dtxE is not a potent elicitor of apoptotic and necrotic cell death. Likewise, in resistant cells only at a 50-fold higher amount of dtxE, an insignificant trend to increased apoptosis rates was found (Figure 1B, right panel). In addition, mitochondrial membrane depolarization due to destruxin treatment was only observed in parental cells after dtxA, B and E incubation, while no significant increase was detected in any of the three resistant sublines (Figure 1C).

HCT116/dtxA and HCT116/dtxB cells were markedly cross-resistant to each other with 18.2- and 21.8-fold higher IC_50_ values than the parental cells, respectively (Table 1). In addition, both sublines showed distinct cross-resistance towards dtxE with tolerance levels matching or even exceeding the one for the dtxE-selected subline. Surprisingly, however, we discovered a collateral sensitivity of dtxE-resistant cells against dtxA and dtxB with a relative resistance factor of 0.3 in the cell viability assay for both resistant sublines (Table 1).

### Mechanisms of destruxin resistance

#### Resistance of HCT116 cells to dtxA, dtxB or dtxE is not mediated by ATP-binding cassette drug efflux transporters

**Figure 2:**
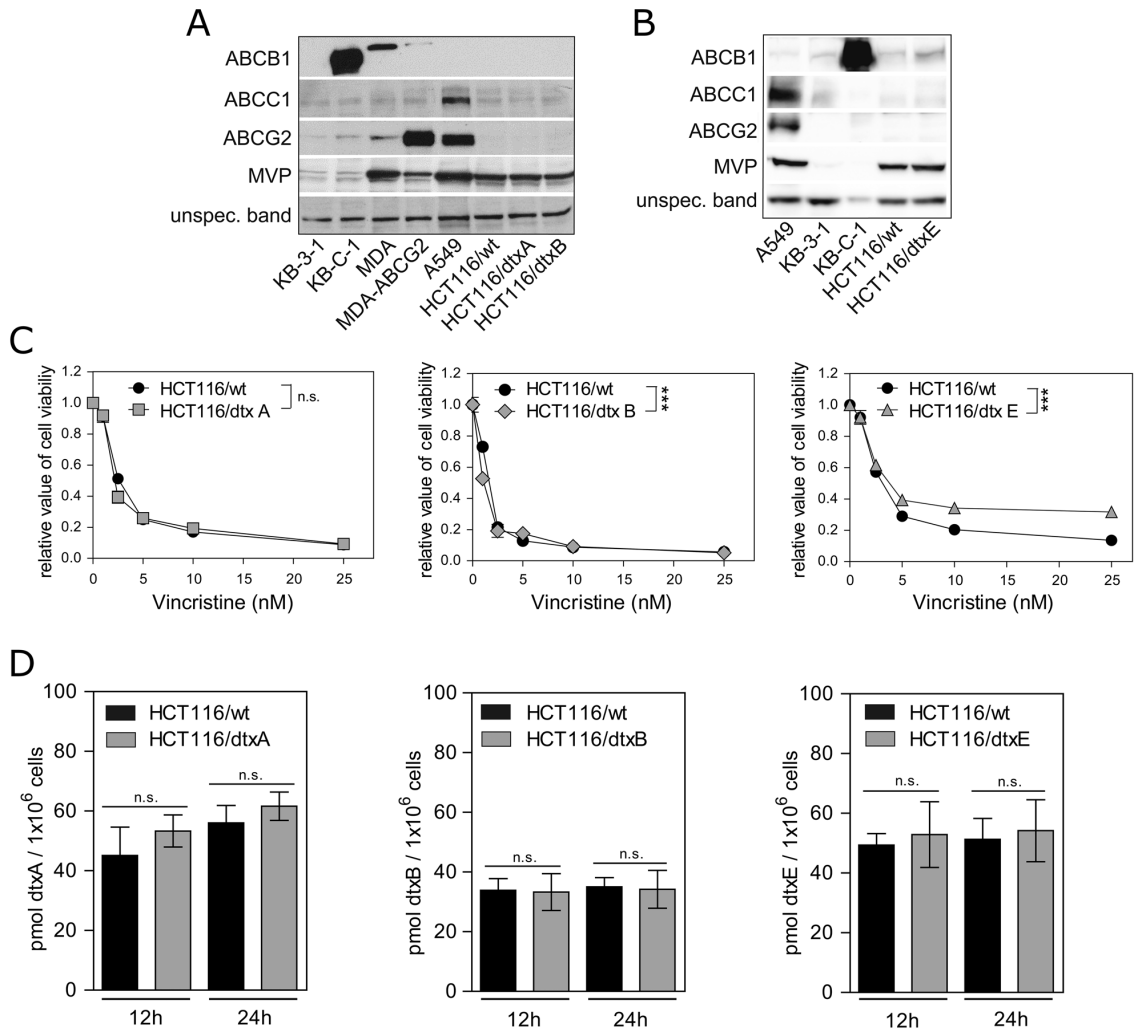
Expression of selected resistance-mediating proteins in parental and dtx-resistant cells ABCB1, ABCC1, ABCG2 and MVP expression levels membrane protein-enriched fractions of **(A)** dtxA-, and dtxB- as well as **(B)** dtxE-resistant sublines were compared to parental HCT116/wt cells. (A, B) As respective controls for resistance protein expression parental MDA cells and ABCG2-overexpressing MDA-ABCG2 cells, parental KB-3-1 and ABCB1-overexpressing KB-C-1 cells as well as ABCC1- and ABCG2-overexpressing A549 cells were employed. **(C)** Cell viability of parental cells (HCT116/wt) and cells of the sublines HCT116/dtxA (left), HCT116/dtxB (middle) and HCT116/dtxE (right) treated with the indicated concentrations of vincristine for 72 h are presented. One representative out of three independent experiments is shown. **(D)** Cellular accumulation of dtxA (left), dtxB (middle) and dtxE (right) in HCT116/wt cells as compared to the respective destruxin-resistant sublines were determined after incubation with 20 μM of the corresponding destruxin for 12 h and 24 h. Mean values (± SD) from three independent experiments are given.

As resistance to chemotherapeutics is frequently mediated by increased expression of ATP-binding cassette (ABC) drug efflux transporters [54] or resistance mediating proteins such as the major vault protein (MVP) [55], we investigated the expression of ABCB1, ABCC1, ABCG2 and MVP in resistant sublines compared to the parental cells. As positive controls, ABCB1-overexpressing KB-C-1 and MDA-MB-231 (MDA) cells, and ABCG2-positive MDA (MDA-ABCG2) and A549 cells were used (Figure 2A) [56, 57]. However, expression of these two transporters was detected neither in the parental HCT116 cells nor in the dtxA- or dtxB-resistant sublines. In case of ABCC1 and MVP, comparably low expression levels of these resistance proteins were observed in the parental and in both selected cell lines (Figure 2A). Correspondingly, mRNA expression levels for *ABCB1*, *ABCC1*, *ABCC2*, *ABCG2* and *MVP* were not altered in HCT116/dtxA cells compared to the parental control. Solely for the dtxB-resistant cells a lower expression of *ABCB1* and *MVP* mRNA was found, which could not be confirmed in the Western blot analyses (Figure 2A, Supplementary Figure 1). For the dtxE-resistant cells similar protein expression levels compared to parental cells were detected for ABCC1, ABCG2 and MVP, while a minor but clear increase of ABCB1 was observed (Figure 2B). Accordingly, mRNA levels of *ABCB1* were significantly increased in HCT116/dtxE cells compared to parental cells, whereas for *ABCC1*, *ABCC2* and *ABCG2* no increase was detected (Supplementary Figure 1). Surprisingly, also *MVP* mRNA levels were significantly increased. Additionally, vincristine, an ABCB1 substrate [58], showed a significantly reduced cytotoxic activity in resistant HCT116/dtxE cells (Figure 2C, right). This effect was absent or very weak in case of HCT116/dtxA and HCT116/dtxB cells, respectively (Figure 2C, left and middle). Hence, these data suggest a functional relevance of the increased ABCB1 expression in dtxE-resistant cells (Figure 2C). Moreover, the sensitivity of HCT116/dtxE cells towards dtxE could be partly restored by co-incubation with ABCB1 inhibitors, i.e. verapamil and cyclosporine A (CSA) [59] (Supplementary Figure 2). However, intracellular destruxin levels remained unchanged in all respective resistant cell lines including HCT116/dtxE after 12 h and 24 h of drug incubation (Figure 2D). These results are unexpected considering the functional impact of increased ABCB1 levels detected in the dtxE subline (Figure 2B, Figure 2C right panel, Supplementary Figure 1). This suggests that the ABCB1-mediated protection against dtxE is not based on potent drug efflux but probably on altered subcellular drug sequestration.

### Cholesterol biosynthesis is upregulated in the destruxin-resistant sublines

**Figure 3:**
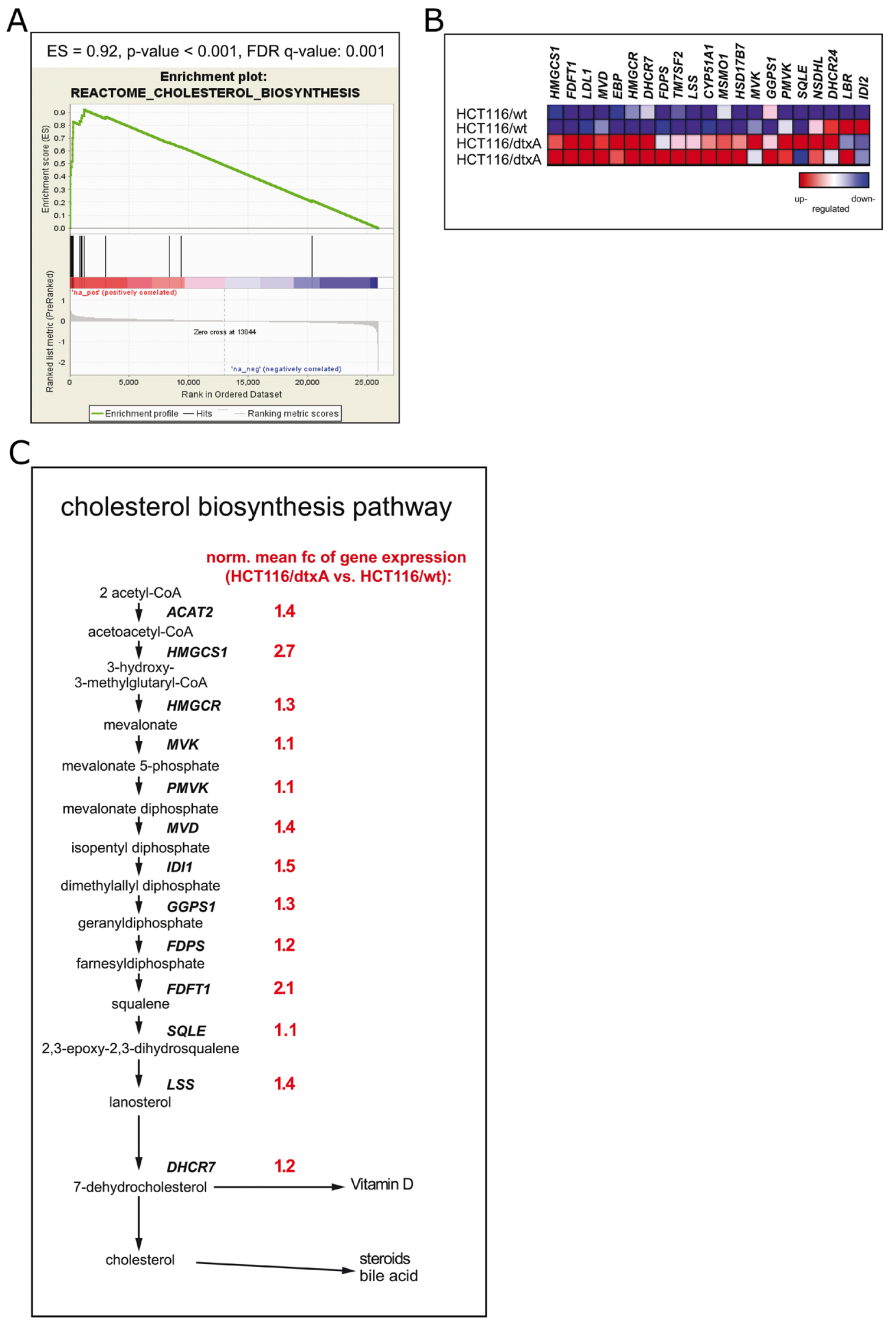
Hyperactivation of the cholesterol synthesis pathway in HCT116/dtxA cells **(A)** Gene set enrichment analysis (GSEA) results concerning the Reactome_Cholesterol_Biosynthesis gene set are depicted: enrichment score (ES), p-value and false discovery rate value (FDR) are listed above the enrichment score curve (green). The gray waterfall plot at the bottom of the chart represents all genes of the human genome which were ranked (x-axis) from highest (left) to lowest (right) fold change of mRNA levels (y-axis: log_2_ of fold change) of the resistant HCT116/dtxA cells compared to the parental HCT116/wt cell line. Vertical black lines indicate the rank position of each gene of the Reactome_Cholesterol_Biosynthesis gene set. Red color highlights overexpression, blue color lower gene expression of the resistant compared to the parental cells. **(B)** Heat map displaying differences in gene expression levels of the “Reactome_Cholesterol_Biosynthesis” gene set in HCT116/dtxA as compared to parental HCT116 cells indicated by blue color for reduced and red color for enhanced gene expression. **(C)** Relative values of gene expression for enzymes of the mevalonate/cholesterol synthesis pathway in HCT116/dtxA compared to parental cells are listed in red alongside the respective gene symbols. Mean -fold gene expression changes derived from normalized values of two independent RNA isolation experiments are depicted.

**Figure 4:**
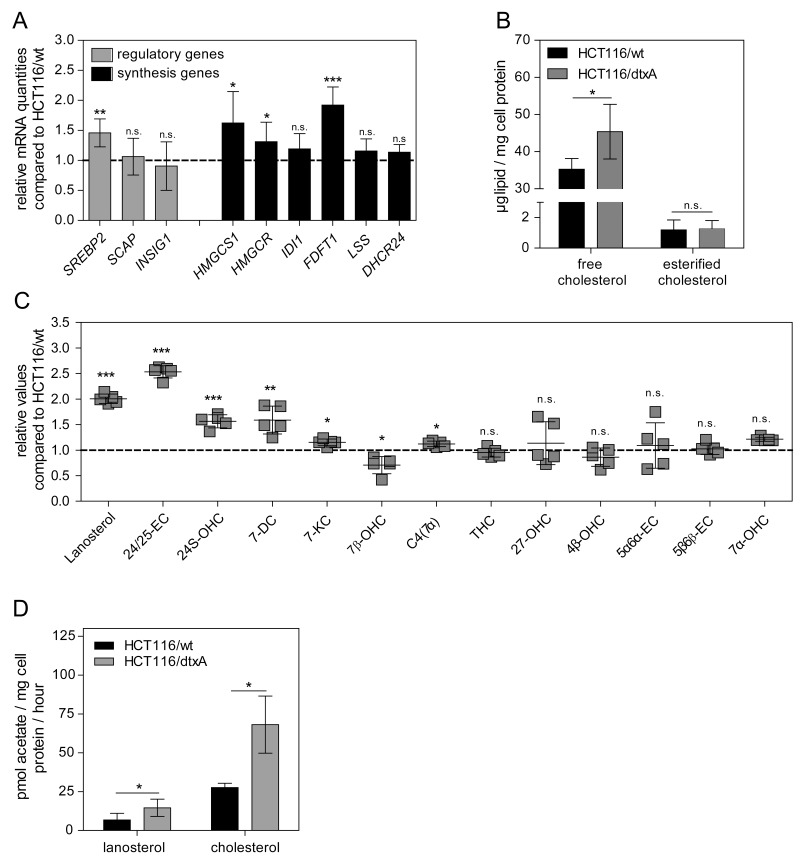
Cholesterol synthesis is upregulated in HCT116/dtxA cells **(A)** mRNA expression levels of selected genes involved in regulation and synthesis of cholesterol, normalized to the housekeeping gene *ACTB*, are expressed as relative values compared to the parental cell line HCT116/wt (dashed line). Results are given as mean values from two experiments with each sample analyzed in duplicates by qRT-PCR. **(B)** Levels of free and esterified cholesterol in the parental and dtxA-resistant HCT116 cells were determined by gas chromatography. Means (± SD) of three independent experiments are given. **(C)** Lanosterol and selected oxysterols were determined via LC-MS/MS. Data of a single experiment, carried out in five replicates, were normalized to the respective levels of the parental HCT116/wt cell line (dashed line) and are shown as scatter plot with error bars (mean values ± SD). **(D)** Parental and dtxA-resistant cells were incubated with radioactively labeled acetate. Levels of *de novo* synthesized lanosterol and cholesterol were determined via thin layer chromatography. Means (± SD) of two independent experiments, performed in duplicates, are depicted. ^*^p<0.05; ^**^p<0.01; ^***^p<0.001.

As overexpression of ABC drug efflux transporters did not account for destruxin resistance, cross-resistance of all three sublines to various chemotherapeutic drugs as well as other fungal metabolites was investigated. No significant cross-resistance of any destruxin-insensitive subline was detectable (Supplementary Tables 6, 7). Likewise, genome-wide array comparative genomic hybridization (aCGH) analysis revealed no distinctive gene-dose alterations at the genomic DNA level in any resistant subline (data not shown). Therefore, to discover possible biological pathways mediating destruxin-resistance, genome-wide gene expression array data were analyzed by GSEA (Supplementary Table 3) [60]. In the dtxA- (Figure 3A) and dtxB- (Supplementary Figure 3A, left panel) but not in the dtxE-tolerant subline (data not shown), GSEA clearly revealed the “Reactome“ pathway “Cholesterol_Biosynthesis” as the top-ranking gene set with an enrichment score (ES) of 0.92 and 0.77 and a false discovery rate (FDR) of <0.001 and 0.006 for the two sublines, respectively. Likewise, the “Steroid_Biosynthesis” and “Terpenoid_Backbone_Synthesis” gene sets from the KEGG database, known to be associated with cholesterol synthesis, were identified with significant enrichment scores and low false discovery rates in our analyses (shown for HCT116/dtxA in Supplementary Figure 3B, Supplementary Figure 3C left panels). The z-transformed expression levels of all genes of the respective gene sets are depicted as heat maps, indicating upregulated gene expression in dtxA-resistant cells in red and downregulated gene expression in blue. Clearly, expression of almost all cholesterol biosynthesis genes concerning the mevalonate pathway was distinctly upregulated in HCT116/dtxA cells (Figure 3B; Supplementary Figure 3B and Supplementary Figure 3C, right panels). An overview of the cholesterol biosynthesis pathway with the respective fold-changes (fc) (i.e. expression values of dtxA-resistant divided by those of parental cells) is given in Figure 3C, indicating an upregulation of gene expression ranging from 10% (*SQLE, PMVK, MVK*) to 270% (*HMGCS1*). As comparable upregulation of the endogenous cholesterol synthesis pathway was found in the dtxA- and the dtxB-resistant models paralleled by strong cross-resistance between the two destruxins, further analyses focused on the highest-resistant HCT116/dtxA cell model. In agreement with array-based gene expression data, qRT-PCR revealed significantly increased mRNA levels for *HMGCS1* (1.6-fold), *HMGCR* (1.3-fold) and *FDFT1* (1.9-fold) and trends towards elevated levels of *IDI* and *LSS*, respectively in the dtxA-resistant subline (Figure 4A, black bars). Although expression of the cholesterol pathway regulatory genes *SCAP* and *INSIG1* did not differ significantly, *SREBP2*, the main transcriptional regulator of cholesterol synthesis genes [61], was found to be significantly increased by 40% in dtxA-resistant cells (Figure 4A, gray bars).

Direct quantification of intracellular lipid levels by gas chromatography in HCT116/wt and the dtxA-resistant subline revealed a distinct increase of free cholesterol by approximately 30% in resistant cells, whereas for the storage form, i.e. esterified cholesterol, similar concentrations were detected in both cell lines (1.2 ± 0.7 and 1.2 ± 0.6 μg lipid/mg cell protein, respectively, Figure 4B). Similar to free cholesterol, also the base level of lanosterol, a precursor of cholesterol, was increased 2-fold in the resistant compared to parental cells (Figure 4C). Additionally, concentration of five oxysterols (24/25-EC, 24S-OHC, 7-DC, 7-KC, and C4(7 α)), i.e. oxidized derivatives of cholesterol and intermediate forms required for the synthesis of steroid hormones, bile acids or vitamin D [62], were found to be significantly increased between 1.1-fold (C4(7 α)) and 2.5-fold (24/25-EC), while 7β-OHC was significantly decreased (Figure 4C). Experiments on cholesterol synthesis rates by detecting incorporation of radiolabeled acetate demonstrated a significant increase of lanosterol (2.1-fold) and cholesterol synthesis (2.5-fold) in dtxA-resistant cells (Figure 4D). Altogether, this suggests that destruxin resistant cells display an elevated expression of cholesterol biosynthetic enzymes leading to enhanced cholesterol biosynthesis and increased levels of cholesterol and its derivatives.

### Pharmacological inhibition of cholesterol biosynthesis attenuates acquired dtxA resistance

**Figure 5:**
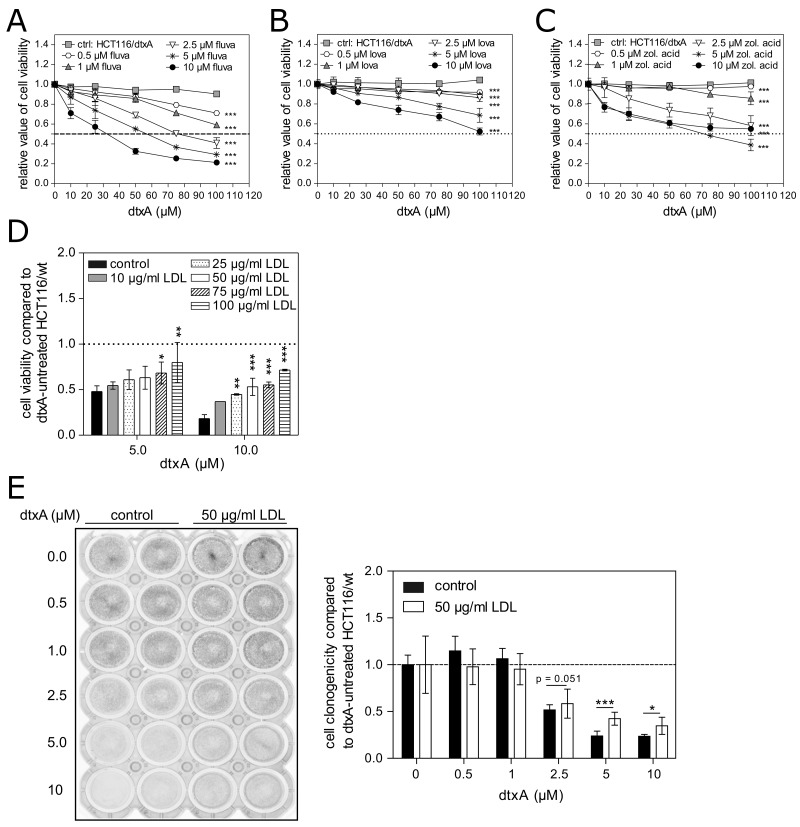
Impact of altered cellular cholesterol levels on acquired resistance and intrinsic sensitivity against DtxA Cells were pretreated with different concentrations of the mevalonate pathway inhibitors **(A)** fluvastatin, **(B)** lovastatin and **(C)** zoledronic acid for 24 h. Subsequently, the indicated concentrations of dtxA were added for another 72 h, and mean (± SD) cell viability normalized to the respective controls, are shown from one representative experiment performed in triplicates. 50% inhibition of cell viability is indicated by dashed lines. **(D)** Parental HCT116/wt cells were left untreated or treated with increasing concentrations of LDL without or with dtxA as indicated for 72 h in growth medium containing 1% FBS. Cell viability values are given relatively to the respective experimental groups without dtxA all set to 1 (dashed line) and are shown as mean (± SD) of three independent experiments performed in triplicate. **(E)** A representative image (left panel), illustrating clonogenic cell growth of HCT116/wt cells treated with increasing concentrations of dtxA as indicated without (left; control) or with 50 μg/mL LDL (right). Darker wells comprise a higher number of viable cell clones. Evaluation of two independent experiments as the one shown on the left side, each performed in duplicates (right panel). Results are given normalized to the respective groups without dtxA and are depicted as means (± SD). ^*^p<0.05; ^**^p<0.01; ^***^p<0.001.

The functional relevance of increased cholesterol synthesis supporting resistance to dtxA was tested by co-incubation with two different classes of mevalonate pathway inhibitors, in order to block cholesterol synthesis at different steps: 1) fluvastatin or lovastatin targeting HMGCR, the rate-limiting enzyme in cholesterol biosynthesis [63]; 2) zoledronic acid, an aminobisphosphonate targeting several enzymes of the mevalonate pathway including farnesylpyrophosphate synthase and geranylgeranyl pyrophosphate synthase [64]. Treatment of parental as well as dtx-resistant cells with these cholesterol inhibitors revealed that especially the dtxA-selected subline showed moderate but distinctive cross-resistance against mevalonate pathway inhibition (Supplementary Figure 4A-4C). Also dtxA sensitivity of resistant HCT116 cells was concentration-dependently enhanced by treatment of cells with both inhibitor classes (Figure 5A-5C). At 2.5, 5 and 10 μM, both statins exerted massive synergistic effects (Figure 5A, 5B). This was verified by calculation of combination indexes (CI) [53] distinctly smaller than 1 at fluvastatin and lovastatin concentrations above 2.5 μM in combination with dtxA (Supplementary Figure 5A, 5B). In line with the results obtained with statins, treatment with zoledronic acid significantly attenuated dtxA resistance with all CI values clearly below 1 (Figure 5C, Supplementary Figure 5C). In addition, the cytotoxic activity of dtxA was augmented by fluvastatin, lovostatin and zoledronic acid in parental HCT116 (Supplementary Figure 6A-6C), breast cancer MCF7 (Supplementary Figure 6D, 6E) and neuroblastoma BT20 (Supplementary Figure 6F, 6G) cells, where the latter two models have an intrinsically low sensitivity to this cyclic peptide. These data suggest that increased cellular cholesterol synthesis via the mevalonate pathway is functionally involved in acquired as well as intrinsic dtxA resistance.

### External cholesterol supplementation reduces dtxA cytotoxicity

To further verify that the increased cellular cholesterol levels downstream of the mevalonate pathway are drivers of the resistance mechanism, we investigated if dtxA-sensitive cells can be protected from dtxA-mediated cytotoxicity by increasing cellular cholesterol levels. Therefore, HCT116 parental cells were supplied with increasing quantities of LDL-cholesterol, the main transport form of cholesterol in human plasma [65], in combination with various concentrations of dtxA. Incubation of dtx-sensitive HCT116/wt cells with LDL alone resulted in a reduced cell viability (Supplementary Figure 7), however dtxA-treated cells were up to 3.9-fold more viable when treated with LDL compared to cells treated with dtxA alone (Figure 5D). Additionally, the clone forming capacity of parental HCT116 cells treated with dtxA in combination with 50 μg/ml of LDL was increased by 50% compared to cells incubated with dtxA only (Figure 5E). These experiments clearly verify that increased cellular cholesterol levels mediate resistance against dtxA.

### DtxA-resistant cells exhibit altered membrane characteristics affecting the pore forming activity of dtxA

**Figure 6:**
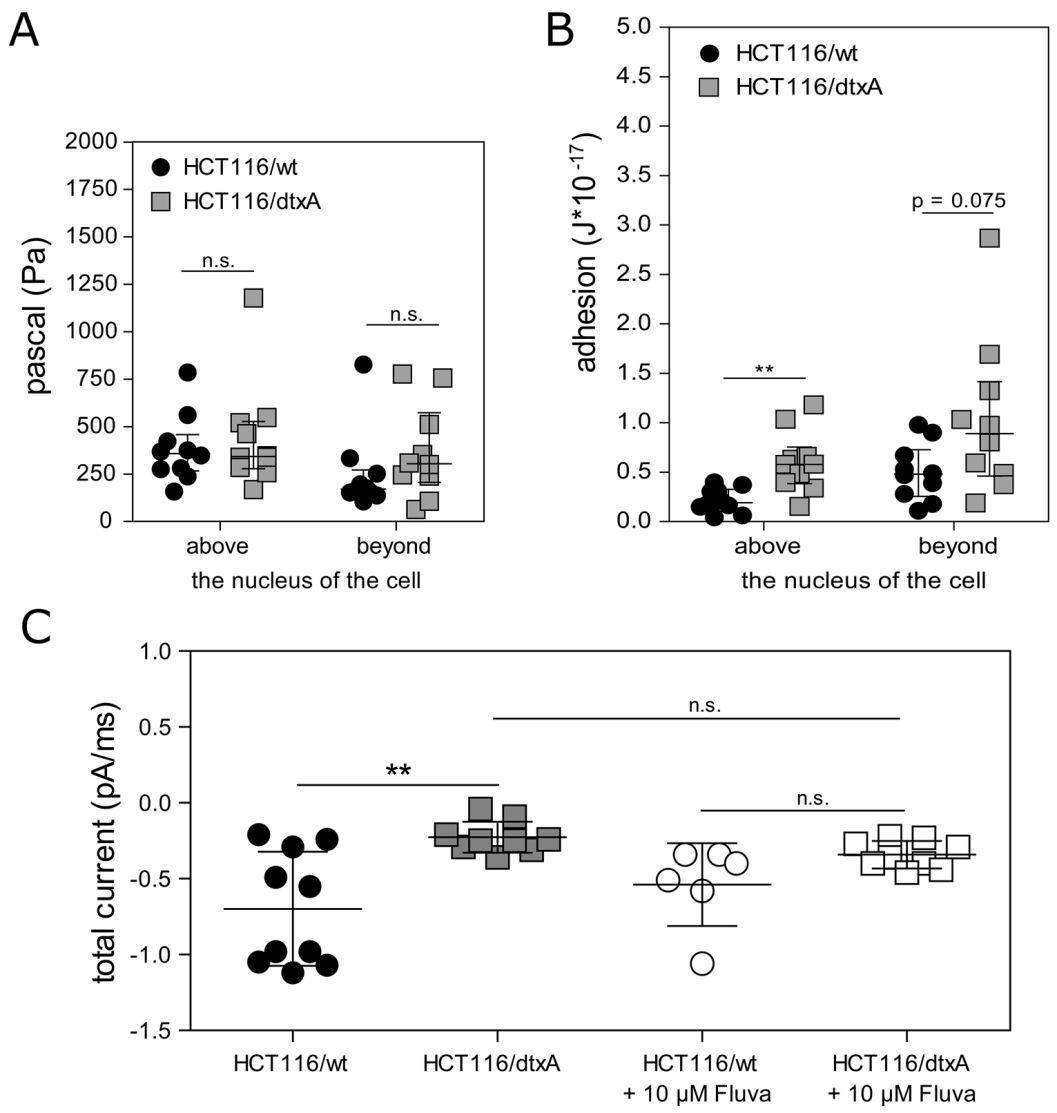
Altered membrane characteristics and reduced pore forming activity of dtxA in HCT116/dtxA cells **(A)** The elastic response of cell membranes was determined by AFM and results of ten measurements above and beyond the nucleus of parental and dtxA-resistant cells are shown as scatter plots with median ± interquartile range. **(B)** Adhesion of cell membranes to the cantilevers above and beyond the nucleus of the parental and dtxA-resistant cells was determined (n=10) and values are shown as scatter plot with median ± interquartile range. **(C)** HCT116/wt and HCT116/dtxA cells were incubated with 20 μM of dtxA and the ionic current was measured by patch clamp technique in whole-cell mode at a membrane potential of -60 mV. Parental cells and dtxA-resistant cells were pretreated with 10 μM fluvastatin as indicated for 24 h prior to dtxA incubation. Results of individual measurements are depicted as scatter plots with error bars (mean ± SD). ^**^p<0.01.

To investigate if, due to increased cholesterol levels, biomechanical properties of the membrane of dtxA-resistant cells were altered, we performed atomic force microscopy experiments. In terms of elastic deformation of the cell membrane above or beyond the nucleus, no differences were observed between resistant and parental cells (Figure 6A). However, a clear difference between the two cell lines was detected in their ability to adhere to the cantilever: in resistant cells, on average a two- to three-fold force had to be applied to detach the cantilever from the center or the periphery of the cells, respectively (Figure 6B). In the context of altered membrane properties in dtxA-resistant cells as described above and due to the fact that an ionophoric activity for destruxins was suggested [37], we tested if the ionophoric effects of dtxA were changed in resistant compared to parental cells. Indeed, in the resistant cells the pore forming ability of dtxA was significantly and distinctly diminished (from a mean total current of -0.7 pA/ms in parental cells to -0.2 pA/ms in the resistant subline) (Figure 6C). After pre-incubation of both cell lines with 10 μM fluvastatin, the significant difference between the two cell lines was completely lost (Figure 6C). Altogether, this suggests a protective effect of membrane cholesterol by diminishing the ionophoric capacity of dtxA in resistant cells.

## DISCUSSION

Destruxins are fascinating bioactive compounds of natural origin which exert, besides insecticidal and antiviral activities, potent anticancer effects [19, 20, 28, 36, 66]. While several molecular modes of action, including ionophoric functions [37] and V-ATPase inhibition [39, 40], have been elucidated, cellular protection mechanisms against destruxins are widely unknown. In the present study, we explored - for the first time - molecular processes underlying resistance of colorectal cancer cells to destruxins. Resistance to chemotherapeutic agents is akin to an evolutionary process within tumors with positive selection of those cell clones either intrinsically insensitive or able to acquire resistance to the applied drug [67]. In the last decades, manifold kinds of resistance mechanisms have been elucidated. Resistance was shown to be mediated by inhibition of apoptosis induction, (e.g. due to increased survival signals) or by metabolization of the active compound, epigenetic alterations, mutations of drug targets, as well as enhanced efflux through different ABC drug transporters [68].

In our hands, colorectal cancer cells readily developed acquired resistance against the three most prevalent destruxins based on a stepwise selection process. Hence, we were able to induce a substantial (i.e. > 50-fold) resistance of HCT116 cells to dtxA, B and E within one year of selection against the respective drug isoform. Interestingly, dtxA and dtxB-resistant cells were highly cross-resistant to all three destruxin derivatives, whereas dtxE-selected cells became collateral sensitive to dtxA and dtxB. These results are suggestive of different factors mediating dtxE as compared to dtxA/dtxB resistance. Interestingly, we did not observe any significant cross-resistance to a number of widely used anticancer compounds as well as other naturally-occurring cyclic depsipeptides which are structurally related to destruxins such as beauvericin, enniatin B and bassianolide. This argues for unique mechanisms of resistance affecting this group of mycotoxins possibly reflecting their complex and interesting mode-of-action different from other better characterized cyclohexadepsipeptides [19, 66]. Concerning previous studies, a broad range of resistance levels was reported for several cyclic depsipeptides depending on selection conditions and the cell type used. For romidepsin, a cell line dependent resistance factor of 7 to 1700 was observed after one month of selection [69]. In contrast, for enniatin only a 2-fold and for beauvericin even no resistance was obtained after an 18 months selection period of a HeLa cell derivative [70]. However, despite considerable differences in the resistance levels induced, overexpression of various types of ABC transporters were involved in all these resistance phenotypes [69, 70]. Our first attempts to unravel mechanisms of resistance to destruxins were therefore targeted at efflux transporters.

Unexpectedly, although all destruxins were found to be substrates of ABC transporters (manuscript in preparation), no increase of any respective mRNA or protein was detectable in both dtxA- or dtxB-selected colon carcinoma cells. In contrast, moderate induction of the ABCB1 gene expression was selectively found in dtxE-resistant cells. The functional relevance of the latter observation was corroborated by a minor but significantly decreased responsiveness of dtxE-resistant cells against the ABCB1 substrate vincristine [58]. Furthermore, sensitivity to dtxE was partly restored by the ABCB1 inhibitors verapamil and CSA [59]. Hence, we concluded that the pronounced resistance of colorectal carcinoma cells to dtxA and B was not based on the activity of drug efflux transporters. These might, nevertheless, play a minor role in resistance to dtxE. Surprisingly, however, intracellular concentrations of all investigated destruxins including dtxE were comparable in the parental and the respective resistant sublines. Similar results in terms of reduced sensitivity together with unaltered drug accumulation rates were obtained for beauvericin and enniatin in ABC transporter-overexpressing cells [70]. One explanation for this unexpected result might be that transmembrane ABC transporters may interact with the ionophoric activity of dtxE and other cyclohexadepsipeptides in the cell membrane. Additionally, as suggested for other compounds [71], ABC transporters might pump dtxE into subcellular compartments (e.g. lysosomes), preventing them from interaction with their intracellular targets. Furthermore, we observed that dtxE-resistant cells were hypersensitive to dtxA/B treatment. These data further indicate that dtxE exerts another mode-of-resistance compared to dtxA or dtxB. Probably the epoxide ring, solely in the structure of dtxE, leads to participation of reactive oxygen species (ROS) in the anticancer activity of this derivative as has been demonstrated by our group in Dornetshuber et al. [20]. So obviously, one of the changes leading to acquired resistance against dtxE renders cells vulnerable to dtxA/B. We can exclude the slight overexpression of ABCB1 in the HCT116/dtxE subline, as other cell models overexpressing this drug transporter are rather resistant against all destruxins including dtxA and dtxB (manuscript in preparation). So, the additional, probably ROS-dependent, factor in the HCT116/dtxE subline rendering it hypersensitive to dtxA/B will be addressed in upcoming projects. In addition, further investigations are needed to clarify the specific interaction of ABC transporters with lipophilic mycotoxins and especially those exerting their cytotoxic activity at least in part by their ion pore-forming capacity at the plasma membrane.

As induction of ABC-transporters did not account for the acquired tolerance at least in dtxA- and dtxB-resistant cells, we expanded our investigations of these two resistance models to genome-wide gene expression analyses followed by GSEA. This unsupervised approach gave strong indications for hyperactivation of the mevalonate pathway. This multistep biochemical reaction cascade accomplishes, besides cellular cholesterol synthesis, also the generation of steroid hormones in steroidogenic cells. Additionally, it produces isoprenoids important for prenylation and in turn membrane tethering of members of the small GTPase superfamily such as the Ras oncogene [72]. Although the increase at the single gene level was comparably moderate, enrichment factors in GSEA were high (up to 0.92) for multiple cholesterol associated GO-terms, based on the fact that the majority of mevalonate pathway genes was upregulated in the resistant sublines. This coordinate expression of multiple genes in the cholesterol biosynthesis pathway is in agreement with the fact that most of these genes share common regulation by the transcription factor SREBP-2 (see below). As already suggested by others, a moderate increase of gene expression throughout a whole pathway might be biologically more relevant than a strong increase of expression of only one single gene [60]. This suggests endogenous cholesterol synthesis to be significantly hyper-activated in dtxA- and dtxB- but not dtxE-resistant sublines. This was further supported by higher amounts of lanosterol and free cholesterol as well as *de novo* synthesized lanosterol and cholesterol in dtxA-resistant compared to parental cells. Furthermore, we also detected enhanced levels of five oxysterols (24/25-EC, 24S-OHC, 7-DC, 7-KC, C4(7 α)), the oxidation products of cholesterol in dtxA-resistant cells. Oxysterols have been suggested to participate in cancer development and progression by influencing diverse signaling pathways, inflammation regulation or reactive oxygen species formation as well as resistance development [73]. Solely the 7β-OHC level was significantly reduced in dtxA-resistant cells. Interestingly, this metabolite reduced cell growth and promoted apoptotic cell death in colon carcinoma cells [74]. Hence, the reduction of this inhibitory oxysterol in dtxA-resistant cells might further contribute to the enhanced survival capacity of resistant cells.

Enhanced cholesterol synthesis has been repeatedly associated with the malignant phenotype *per se* and with therapy resistance in particular before. Cancer patients with a hyper-activated cholesterol pathway in the tumor cells might have reduced overall survival rates [75]. It was thus hypothesized that cancer cells are dependent on higher cholesterol levels to support their constant growth and increased membrane synthesis rates [76]. These findings were complemented by several studies showing that intake of statins to block cholesterol synthesis via the mevalonate pathway, was associated with a reduced risk of cancer [77]. Concerning therapy resistance, enhanced cholesterol pathway activity has been reported in cancer cell models resistant to e.g. platinum drugs or doxorubicin [78–83]. Accordingly, also in our experiments dtxA sensitivity could be significantly restored by blockade of the mevalonate pathway by both statins and zoledronic acid, while cholesterol supplementation of destruxin-sensitive parental cells enhanced cell viability. Moreover, significant sensitization to destruxins by mevalonate pathway inhibition was also observed in cell models intrinsically resistant against these mycotoxins. This clearly indicates that cholesterol-involving mechanisms are operative in both intrinsic and acquired resistance to destruxins and, hence, might represent a so far underestimated protection mechanism against diverse natural toxins.

Next, we investigated which mechanism might underlie the broad - though moderate - overexpression of almost all genes in the mevalonate pathway. Therefore, we determined expression levels of key mevalonate pathway regulatory genes, namely *SREBP2*, *SCAP* and *INSIG1*. Out of these, only *SREBP2* - an essential transcription factor governing most genes of the cellular cholesterol homeostasis – was found significantly upregulated in the dtxA-resistant cell model. In its inactive form SREBP2 is bound to SCAP, the cholesterol-sensing protein in the membrane of the endoplasmic reticulum (ER), and to INSIG1, a protein retaining the complex in the ER membrane. At low levels of cholesterol in the ER membrane, SREBP2 and SCAP relocate to the Golgi apparatus where SREBP2 dissociates from SCAP and subsequently translocates into the nucleus where it activates numerous genes of the cholesterol biosynthesis pathway [84]. In addition, SREBP-2 activates its own expression, constituting a positive auto-regulatory mechanism [85]. Elevated SREBP-2 mRNA levels as well as elevated levels of multiple genes involved in cholesterol synthesis are thus indicative of elevated SREBP-2 activity. Possible explanations for SREBP2 hyper-activation might be dtxA-induced upregulation of the Akt/mTOR/S6 pathway or endoplasmic reticulum stress [75, 86]. However, our analysis neither showed increased phosphorylation levels of those proteins nor an increased expression of ER stress-related mediators (data not shown). Hence, the mechanisms of how dtxA activates SREBP2 needs to be elucidated in upcoming projects.

Finally, the question remained which mechanisms mediate the protective effect of cholesterol against destruxins. So far, we could demonstrate that in dtxA-resistant cells biomechanical membrane features were altered. While stiffness was widely unchanged, adhesiveness of the cell membrane to the cantilever (based on, e.g. van der Waals forces and electrostatic forces constituting the overall surface charge in AFM [87]), was significantly enhanced in dtxA-resistant cells. In accordance, cholesterol was previously identified to modify the surface charge of membranes [88, 89]. Additionally, the lipid composition of cellular bio-membranes was demonstrated to be altered in other multidrug resistant cell models [78, 90]. This might be based on the fact that increased cholesterol levels mediate a higher degree of orientational order of phospholipids resulting in a laterally more condensed membrane with increased mechanical strength and decreased permeability [91] for small molecules such as glucose or for gases, water and ions [92]. Correspondingly, a very recent publication proposed that ion channel functions of ionophores are also influenced by cholesterol-rich membrane domains [93]. In agreement, in the dtxA-resistant subline dtxA was unable to induce an ion current comparable to the one in parental cells. This clear-cut difference was lost in the presence of fluvastatin suggesting endogenous cholesterol synthesis and, in turn, altered membrane permeability to be major contributors to acquired destruxin resistance.

## MATERIALS AND METHODS

### Chemicals

Secondary metabolites dtxA, dtxB, and dtxE were obtained from a *M. brunneum* culture broth and purified as previously described in detail [41]. Stock solutions were prepared in DMSO (Sigma-Aldrich, St. Louis, USA) and stored at -20°C. All other inhibitors and solvents used are listed in “Supplementary Table 1”.

### Cell culture

The human colorectal carcinoma cell line HCT116 was kindly provided by Dr. B. Vogelstein (John Hopkins University, Baltimore, USA). BT20 (HTB-19) and MCF7 (HTB-22) cell lines were obtained from ATCC. HCT116 cell lines were maintained in McCoy’s medium with 2 mM glutamine (Sigma-Aldrich). BT20 as well as MCF7 cells were grown in Eagle’s minimum essential medium with 0.01 mg/ml insulin (Sigma). All culture media were supplemented with 10% fetal calf serum (FCS, PAA, Linz, Austria) and kept at 37°C and 5% CO_2_. Cells were authenticated by DNA profiling and/or by array comparative genomic hybridization (aCGH). In addition, all cell lines were regularly screened for *Mycoplasma* contamination.

### Generation of dtxA-, dtxB- and dtxE-resistant HCT116 cells

To establish three dtx-resistant sublines (HCT116/dtxA, HCT116/dtxB and HCT116/dtxE), HCT116/wt cells were treated with gradually increasing concentrations of dtxA, dtxB or dtxE, starting with subtoxic concentrations of 100 nM for the dtxA- and dtxB- and of 10 nM for the dtxE-resistant subline. Once a comparable cell proliferation rate was observed in sublines as compared to parental cells, concentrations of destruxin were increased, reaching 50 μM in the HCT116/dtxA and HCT116/dtxB and 1.2 μM in the HCT116/dtxE subline. After one year of selection, resistant sublines remained stable upon application of a monthly treatment. Resistance levels were regularly monitored by cell viability assay.

### Cell viability assay

The cytotoxic activity of destruxin alone or combined with diverse inhibitors was determined via MTT assay following the manufacturer’s recommendations (EZ4U, Biomedica, Vienna, Austria) or via crystal violet staining [20]. A detailed description is given in the “Supplementary Material and Methods” section.

### Clonogenicity assay

To determine the clone formation capacity of HCT116/wt cells with or without low density lipoprotein (LDL) in combination with various dtxA concentrations, clone formation assays were performed: Cells were seeded in 24-well plates at 1 × 10^4^ cells/well in growth media with 5% FBS. After 24 h of recovery, cells were treated for 6 days with the indicated drug concentrations alone or in combination with 50 μg/ml LDL in growth media with 1% FBS. LDL was isolated from healthy normolipidemic volunteers by sequential flotation ultracentrifugation [42]. Isolation of LDL from human subjects was approved by the ethics committee of the Medical University of Vienna (#1414/2016). Informed consent was obtained from all subjects. Cells were fixed with methanol (Merck KGaA, Darmstadt, Germany) for 10 min at 4°C and stained with crystal violet (1 μg/μl in PBS) for 15 min at RT. The fluorescent signal (610/30 nm BP emission filter, 633 laser) of stained cell clones was scanned with a Typhoon Trio (Amersham Bioscienes, Little Chalfont, UK). From the derived images, pixel intensities per well were quantified by Image J 1.50i (NIH, USA). Values were normalized to the dtxA-untreated control.

### Detection of cell death induction by JC-1 and Annexin-V/PI staining

Parental and dtx-resistant cells were seeded (1x10^5^ cells/well in 6-well plates) and treated on the following day for 48 h with the indicated concentrations of dtxA, dtxB or dtxE. Next, the mitochondrial membrane potential (ΔΨm) was measured. In doing so, cells were detached by trypsinization, centrifuged with 300× g for 5 min at 4°C and stained with the fluorescent dye JC-1 (5,5’,6,6’-tetrachloro-1,1’,3’,3’-tetraethyl-benzimidazolylcarbocyanine iodide) for 10 min at 37°C. FACS analysis was performed according to the manufacturer’s instructions (Mitochondrial Membrane Potential detection Kit; Stratagene, La Jolla, CA, USA). To further compare apoptotic changes in the parental and resistant cells, Annexin V-APC/PI stainings were carried out. An overview of the method is given in the “Supplementary Material and Methods” section.

### Immunoblotting

Membrane-enriched fractions were prepared as published [43], separated by SDS-PAGE and transferred onto a PVDF membrane (Bio-Rad, Vienna, Austria) for Western blotting as defined in former studies [44]. All primary antibodies that were used are listed in “Supplementary Table 2”. Secondary horseradish peroxidase-labeled antibodies (anti-rabbit or anti-mouse, Cell Signalling Technology, Beverly, MA) were diluted 1:10 000 in 1 % BSA in TBST (TBS + 0.1%Tween).

### DNA isolation and array comparative genomic hybridization (aCGH)

A brief description is given in the “Supplementary Materials and Methods” part.

### Gene expression array and analysis

RNA was isolated with the Qiagen RNeasy Kit (Qiagen, Hilden, Germany) according to the manufacturer’s protocol. Quantity and quality were verified with an Agilent 2100 Bioanalyzer (Agilent Technologies, Santa Clara, CA, USA). Only samples with a RNA integrity number (RIN) above 9 were used for whole-genome gene expression analysis. Parental HCT116 and the dtxA-resistant cells were analyzed using a 4x44K whole genome oligonucleotide-based gene expression array (Agilent). Labeling and hybridization was carried out according to the manufacturer’s protocol and as described in detail previously [45, 46]. Slides were scanned on a G2505B microarray scanner (Agilent). Feature extraction and data analysis were carried out using the Feature Extraction and GeneSpring software (both Agilent), respectively. For data analysis in GeneSpring, the following parameters were used (Guided Workflow): samples were thresholded to 1, shifted to the 75% percentile, and the baseline was set to the median of all samples. Simultaneously, data were analyzed for differentially enriched gene sets using Gene Set Enrichment Analysis (GSEA v2.2.4, Broad Institute, Inc., Massachusetts Institute of Technology, Cambridge, USA). In the process, microarrays were background subtracted with the LOESS algorithm and quantile normalized using the LIMMA package in R (version 3.32.2 [47]). A first exploratory analysis pointed towards the activation of cholesterol synthesis-related pathways. In a next step, 11 pathways were selected (Supplementary Table 3) from the KEGG, REACTOME and BIOCARTA databases and used for a second GSEA. The gene sets with a FDR < 0.05 were considered to be significantly enriched.

### Cellular uptake of destruxins

DtxA, dtxB and dtxE accumulation in parental HCT116/wt cells and the corresponding destruxin-resistant subline was measured by HPLC. Five million cells were seeded and treated the following day for 12 h or 24 h with 20 μM of the respective destruxin. Then, cells were trypsinized, washed three times with PBS and lysed by repeated (5 times) shock freezing in liquid nitrogen and subsequent thawing. Following centrifugation at 13,500× g for 5 min, 80 μL of the supernatant (cytoplasm) were quantified by HPLC using a Dionex UltiMate 3000 system (Sunnyvale, CA, USA), equipped with a L-7250 injector, a L-7100 pump, a L-7300 column oven (set at 35°C), a D-7000 interface and a L-7400 UV detector (Thermo Fisher Scientific, Waltham, Massachusetts), set at a wavelength of 220 nm. A brief description of the operating conditions for the chromatographic separation is given in the “Supplementary Materials and Methods” section.

### RNA isolation and quantitative real-time PCR (qRT-PCR)

RNA was isolated with Trizol (Invitrogen, MA, USA) according to standard procedures. Next, mRNA was reverse transcribed into cDNA using the RevertAid Reverse Transcriptase (Fermentas, ThermoFisher Scientific, Waltham, MA, USA). Moreover, qRT-PCR (StepOne Plus, Applied Biosystems, Foster City, CA, USA) was performed for relative quantification of target gene expression using TaqMan probes which are listed in “Supplementary Table 4”.

### Gas chromatography

To evaluate the basic levels of free cholesterol and esterified cholesterol, cells were plated in 6 cm culture plates and grown for 48 h to a confluence of 70 – 80 %. Next, cells were washed twice with 5 mL PBS, trypsinized and centrifuged at 300 × g, for 5 min at 4°C. Cell pellets were stored at – 80°C. Lipid extraction, protein determination and gas chromatography were performed as described previously [48].

### Detection of *de-novo* lipid synthesis from acetate

To evaluate the *de-novo* synthesis of cholesterol and lanosterol, cells were seeded in duplicates in 6-well plates and grown for 48 h until reaching 70 – 80% confluency. After the cells were trace-labeled with 1 μCi/ml ^14^C-acetate (Perkin Elmer, Waltham, MS, USA) for one hour, lipids were extracted from cell monolayers using hexane/isopropanol (3/2). Directly before lipid extraction, ^3^H-oleic acid was added as recovery marker to the cell monolayers to allow for compensation of sample loss during lipid extraction and TLC. Cells were lyzed using 0.1 mol/l NaOH and cell protein was quantified using the Bradford methods. Lipid extracts were saponified using aqueous KOH and non-saponified lipids (i.e. cholesterol and lanosterol) were extracted using hexane. Lipid extracts were concentrated and separated by TLC on silica gel sheets (Polygram SIL G; Marcherey-Nagel, Düren, Germany) using CHCl_3_ as solvent. Spots were detected by iodine vapor (Rf values: 0.23 for free cholesterol and 0.35 for lanosterol), excised, and analyzed by liquid scintillation counting. Values were normalized to the recovery marker and cell protein levels.

### Detection of lanosterol and oxysterol levels

3x10^5^ cells/well were seeded in 6-well plates, grown for 48 h and, after media removal, were washed three times with ice cold PBS. Then, cells were directly scraped [49] in 1 mL ice-cold 80/20 methanol/H_2_O solution (MeOH and H_2_O, LC-MS grade, Sigma-Aldrich) with 0.01% Butylhydroxytoluol (BHT) (Sigma-Aldrich), vortexed and centrifuged with 18,000 × g for 5 min at 4°C. The supernatant dried in a vacuum centrifuge (Savant RVT400, Thermo Scientific, Waltham, MA, USA) and stored at -80°C. LC-MS analysis of lanosterol and oxysterols was performed at Biocrates with an in-house assay for oxysterols (Biocrates Life Sciences AG, Innsbruck, Austria). In this process, free oxysterols were extracted from samples with MeOH (Sigma-Aldrich) using the Biocrates Kit filter plate, which was loaded with an internal standard mixture beforehand. The metabolites were determined by UHPLC-MS/MS with multiple reaction monitoring (MRM) in positive mode using a SCIEX API 5500 QTRAP^®^ (SCIEX, Darmstadt, Germany) instrument with electrospray ionization (ESI). The assay had been previously validated according to European Medicines Agency (EMA) guidelines. To normalize the obtained results, protein concentration was determined in the obtained pellets, with a 2D Quant Kit (GE Healthcare, Munich, Germany) as previously published.

### Whole-cell patch clamp

Ionophoric activity of dtxA, dtxB and dtxE was determined by patch clamp experiments, performed as previously described [20, 50]. Briefly, ionic currents were recorded in whole-cell mode at RT. Glass electrodes with a resistance less than 5 MΩ were pulled with a DMZ universal puller and filled with intracellular solution (120 mM CsF, 20 mM CsCl, 5 mM EGTA, 5 mM HEPES; pH 7.4). HCT116/wt or resistant cells were perfused with extracellular solution (140 mM NaCl, 4 mM KCl, 1.8 mM CaCl_2_, 0.75 mM MgCl_2_, 5 mM HEPES; pH 7.4) in a 1 mL chamber. After obtaining a whole-cell configuration, cells were allowed to equilibrate for 5 min before recording any currents and then pulsed to -60 mV for 10 sec pulses. Data were filtered at 5 kHz and digitized at 10 kHz by using digidata 1440A. Traces were averaged and subtracted from control recording, in the presence or absence of 20 μM dtxA, dtxB or dtxE, to observe ionophoric activity.

### Atomic force microscopy

3000 cells were seeded in 3.5 cm dishes and grown in full growth media for 48 h. Cell elasticity and adhesion were determined with a NanoWizard atomic force microscope (AFM) (NanoWizard, JPK Instruments AG, Berlin, Germany) on a Zeiss Microscope Axio Observer. A1 (Carl Zeiss Microscopy, LLC, Thornwood, NY, USA) using the CellHesion methodology (CellHesion 200, JPK Instruments AG, Berlin, Germany). The low stress silicon nitride cantilevers (spring constant: 0.085 N/m, Hydra-All, AppNano, Applied NanoStructures, Inc., Santa Clara, CA, USA) with an uncoated tetrahedral tip (radius of curvature <8 nm) were calibrated as previously described [51]. The force spectroscopy measurements were performed in contact mode with a force up to 1nN. Force-distance curves were recorded individually without contact time at 25°C with a constant loading rate of 5 μm/s. The contact of the tip to the probe was monitored visually with a Zeiss Microscope camera (bright field microscopy, CCD camera). The tip was either placed above the nucleus or the periphery of the cells. For each cell line, ten cells were probed and seven data curves per cell were determined, respectively. The elastic response or Young´s modulus was determined from the approach curve using the data processing software CellHesion^®^ 200 (JPK Instruments AG, Berlin, Germany) following the Hertz model [52]. Additionally, for the characterization of the adhesion properties, the area under the retract curve (AUC) was quantified.

### Statistics and synergism calculations

Data were analyzed using GraphPadPrism 5 software (GraphPad Software, Inc, La Jolla, USA) and are given as mean values ± standard deviation (SD), if not specified otherwise. Two groups were compared by Student’s *t*-test, while comparisons within several groups were done by 1-way Anova followed by a Dunnett’s multiple comparison test or by 2-way Anova with a Bonferroni post-test. For data with a non-parametric distribution a Mann-Whitney test was performed. The synergistic activity expressed by the combination index (CI) between two compounds, was determined by CalcuSyn software (Biosoft, Ferguson, MO, USA) using the method established by Chou and Talalay [53]; synergism: CI < 1, additivity: CI = 1, antagonism: CI > 1.

## CONCLUSIONS

In conclusion, we have for the first time uncovered acquired resistance mechanisms of human cancer cells to destruxins, a group of cyclopeptidic mycotoxins with an interesting activity profile. Hyperactivation of biochemical cholesterol synthesis via the mevalonate pathway resulting in altered membrane properties and a reduced ionophoric activity was identified as a key protection mechanism against destruxins. Therefore, combinational therapies of destruxins with statins might increase anticancer efficacy and reduce resistance development. Besides its potential anticancer but also antiviral activity, destruxins have been widely used as bio-insecticide employing the entomopathogenic fungus *Metarhizium brunneum*. Whether analogous resistance mechanisms as detected in human cancer cells might also be relevant in insect cells is an interesting topic for further transdisciplinary investigations.

## SUPPLEMENTARY MATERIALS FIGURES AND TABLES



## Abbreviations

AFM: atomic force microscopy
ABCB1: ATP-binding cassette (ABC) transporter B1
ABCC1: ATP-binding cassette (ABC) transporter C1
ABCC2: ATP-binding cassette (ABC) transporter C2
ABCG2: ATP-binding cassette (ABC) transporter G2
ATCC: American Type Culture Collection
BHT: butylhydroxytoluol
dtx: destruxin
CI: combination index
FACS: fluorescence-activated cell sorter
FITC: fluorescein isothiocyanate
FLT-3: Fms-like tyrosine kinase-3
JC-1: 5,5’,6,6’-tetrachloro-1,1’,3,3’-tetraethyl-benzimidazolylcarbocyanine iodide
LC/MS: liquid chromatography mass spectrometry
MDR: multidrug resistance
MTT: 3-(4,5-dimethylthiazol-2-yl)-2,5-diphenyltetrazolium bromide
MVP: major vault protein
NMR: nuclear magnetic resonance
qRT-PCR: quantitative real time polymerase chain reaction
fluva: fluvastatin
lova: lovastatin
LDL: low density lipoprotein
PI: propidium iodide
ACAT2: acetyl-CoA acetyltransferase 2
HPLC: high-performance liquid chromatography
HMGCS1: 3-hydroxy-3-methylglutaryl-CoA synthase 1
HMGCR: 3-hydroxy-3-methylglutaryl-CoA reductase
MVK: mevalonate kinase
PMVK: phosphomevalonate kinase
MVD: mevalonate diphosphate decarboxylase
IDI1: isopentenyl-diphosphate delta isomerase 1
GGPS1: geranylgeranyl diphosphate synthase 1
FDPS: farnesyl diphosphate synthase
FDFT1: farnesyl-diphosphate farnesyltransferase 1
SQLE: squalene epoxidase
LSS: lanosterol synthase
DHCR7: 7-dehydrocholesterol reductase
SREBP2: sterol regulatory element binding transcription factor 2
SCAP: SREBF chaperone
DHCR24: 24-dehydrocholesterol reductase
INSIG1: insulin induced gene 1
LDLR: low density lipoprotein receptor
SDS: sodium dodecyl sulfate
PVDF: polyvinylidene difluoride membrane
CSA: cyclosporine A
GSEA: gene set enrichment analysis
KEGG: Kyoto Encyclopedia of Genes and Genomes
24/25-EC: 24,25-epoxycholesterol
24S-OHC: 24S-hydroxycholesterol
7-DC: 7-dehydro cholesterol
7-KC: 7-ketocholesterol
7β-OHC: 7ß-hydroxycholesterol
C4(7α): 7α-hydroxycholestenone
THC: 5α,6ß-dihydroxycholestanol
27-OHC: 27-hydroxycholesterol
4β-OHC: 4β-hydroxycholesterol
5α6α-EC: 5α,6α-hpoxycholesterol
5β6β-EC: 5β,6β-hpoxycholesterol
7α-OHC: 7α-hydroxycholesterol
RIN: RNA integrity number
