# Supplementary material for: Altered membrane rigidity via enhanced endogenous cholesterol synthesis drives cancer cell resistance to destruxins
Source: Oncotarget. 2018 May 22;9(39):25661–80. doi: 10.18632/oncotarget.25432 (PMC5986646; doi:10.18632/oncotarget.25432)
Supplement: Supplementary file 1 [file oncotarget-09-25661-s001.pdf]

Altered membrane rigidity via enhanced endogenous cholesterol synthesis drives cancer cell resistance to destruxins

SUPPLEMENTARY MATERIALS

Material and Methods

Chemicals

The supplier of all inhibitors and the dilution media that were used are listed in “Supplementary Table 1”. DMSO concentrations of all applied drug dilutions did not exceed 1%.

Cell viability assays

In general, 2 × 10^3 cells per well were seeded in full-growth media with 10% FBS into 96-well plates and incubated at 37°C (5% CO_2) overnight. Then, cells were treated with increasing concentrations of dtx or drug compounds for 72 h. For the treatment with fluvastatin, lovastatin or zoledronic acid, 1 × 10^3 cells per well were seeded and after overnight recovery, cells were treated with these compounds alone. After 24 h of preincubation, dtxA was added at the indicated concentrations and then cells were incubated for further 72 h. The percentage of viable cells was detected after incubation with 3-(4,5-dimethylthiazol-2-yl)-2,5-diphenyltetrazolium bromide (MTT) at 37°C for approximately one hour, according to the user manual (EZ4U, Biomedica, Vienna, Austria). The inhibitory concentration (IC_{50}), i.e. reduction of viable cells by 50%, was calculated from whole-dose response curves using GraphPadPrism 5 software (GraphPad Software, Inc, La Jolla, USA). To investigate the protective effect of LDL against the cytotoxic activity of dtxA in HCT116/wt cells, 1 × 10^4 cells per well were seeded in growth media with 5% FBS. After overnight recovery, cells were treated with increasing concentrations of LDL and dtxA for 72 h in growth media with 1% FBS. Cell viability was determined after washing cells once with ice cold PBS, fixing the cells for 10 min with ice cold MeOH (Merck KGaA, Darmstadt, Germany) at 4°C and staining the cells with crystal violet (Sigma-Adrich, 1 µg/µL in PBS) for 10 min at RT. For quantification of viable cells, the crystal violet dye was dissolved in 2% SDS and the absorbance was measured at 520 nm with a TECAN absorbance reader (Tecan Group Ltd., Männedorf, Switzerland). All experiments were conducted in triplicates and repeated three times.

Detection of cell death induction by JC-1 and Annexin-V/PI staining

The parental cell line as well as the three resistant sublines (1 × 10^5/well in 6-well plates) were seeded, incubated overnight at 37°C and treated with increasing concentrations of destruxin. After 48 h of treatment, cells were trypsinized and centrifuged for 5 min at 300xg. Cells were resuspended in 100 µL buffer A (10 mM HEPES, 140 mM NaCl and 2.5 mM $CaCl_2$) and stained for 15 min under light-protection with PI (propidium-iodide, 1 µg/mL, Sigma-Aldrich) and Annexin-V-APC (20 µL/mL, BD Biosciences). Following addition of 200 µL buffer A 10,000 cells were measured by FACS analysis (FACSCalibur; Becton Dickinson) and the percentage of viable cells was determined by CellQuest Pro software (Becton, Dickinson and Company, NY, USA).

Quantification of destruxin accumulation in cells

Chromatographic separation of dtxA, dtxB, dtxE was performed on a Hypersil BDS-C18 column (5 µm, 250 x 4.6 mm Thermo Electron Corporation), preceded by a Hypersil BDS-C18 precolumn (5 µm, 10 x 4.6 mm), at a flow rate of 1 mL/min. The mobile phase consisted of 10 mM ammonium acetate/acetic acid buffer, pH 7.4 (mobile phase A), and acetonitrile (mobile phase B). A linear gradient was applied, to elute dtxA, dtxB and dtxE according to their lipophilicity. The gradient ranged from 10% acetonitrile at 0 min to 90% B at 17 min and linearly increased to 95% B at 18 min at which it remained constant until 25 min. Subsequently, the percentage of acetonitrile was decreased within 2 min to 10% in order to equilibrate the column for 8 min before injection of the next sample. Quantification was accomplished using the external standard method. Linear calibration curves were performed by spiking drug-free cell culture medium with standard solutions of dtxA, dtxB and dtxE to give a concentration range from 0.05 to 10 µg/mL (average correlation coefficients: >0.999). For this method the lower limit of quantification for dtxA, dtxB and dtxE was 0.05, 0.08 and 0.08 µg/mL, respectively. Coefficients of accuracy and precision for both compounds were < 9%.

DNA isolation and array comparative genomic hybridization (aCGH)

DNA of HCT116/wt and all three resistant sublines were isolated using the QIAamp DNA Blood Mini Kit (Qiagen, Hilden, Germany) adhering to the manufacturer’s protocol. aCGH was performed using 4x44K whole genome oligonucleotide-based arrays (Agilent, Santa Clara, CA). Labeling and hybridization procedures were performed according to the instructions provided by Agilent using the SureTag DNA Labeling Kit and as previously published [1, 2].

REFERENCES

1. Mathieu V, Pirker C, Schmidt WM, Spiegl-Kreinecker S, Lotsch D, Heffeter P, Hegedus B, Grusch M, Kiss R, Berger W. Aggressiveness of human melanoma xenograft models is promoted by aneuploidy-driven gene expression deregulation. Oncotarget. 2012; 3:399-413. https://doi.org/10.18632/oncotarget.473.

2. Schmidt WM, Uddin MH, Dysek S, Moser-Thier K, Pirker C, Hoger H, Ambros IM, Ambros PF, Berger W, Bittner RE. DNA damage, somatic aneuploidy, and malignant sarcoma susceptibility in muscular dystrophies. PLoS Genet. 2011; 7:e1002042.

Supplementary Table 1: Description of inhibitors used in this study

Inhibitor	Mode of action	Dilution medium	Supplier
fluvastatin sodium salt hydrate	competitive HMG-CoA reductase inhibitor	ddH_2O	TCI Europe NV (Zwijndrecht, Belgium)
lovastatin (Mevinolin)	competitive HMG-CoA reductase inhibitor	DMSO (Sigma-Aldrich)	Sigma-Aldrich (St. Louis, USA)
zoledronic acid monohydrate (Zometa)	farnesyl diphosphate (FPP) synthase inhibitor	ddH_2O	Sigma-Aldrich (St. Louis, USA)

The solution of all inhibitors diluted with ddH_2O was sterilized by using a Minisart RC 4 sterilizing filter (0.2µm, VWR, Radnor, Pennsylvania, USA).

Supplementary Table 2: Description of antibodies used in this study

Detected protein	Species	Diluted in 3 % BSA in TBST	Company
ABCB1	monoclonal mouse	1:100	Calbiochem (San Diego, CA, USA)
ABCC1	monoclonal mouse (clone MRPm6)	1:1000	MONOSAN® (AM Uden, The Netherlands)
ABCG2	monoclonal mouse	1:500	Chemicon International (Temecula, California, USA)
MVP (LRP)	monoclonal mouse	1:1000	BD Transduction Laboratories (San Jose, CA, USA)

BSA, bovine serum albumin (Roth, Karlsruhe, Germany); TBST, TBS + 0.01%Tween (Bio-Rad);

Supplementary Table 3: Gene sets associated with cholesterol synthesis

Gene set
KEGG_STEROID_HORMONE_BIOSYNTHESIS
REACTOME_CHOLESTEROL_BIOSYNTHESIS
REACTOME_FATTY_ACID_TRIACYLGLYCEROL_AND_KETONE_BODY_METABOLISM
BIOCARTA_AKT_PATHWAY
REACTOME_FATTY_ACYL_COA_BIOSYNTHESIS
BIOCARTA_MTOR_PATHWAY
KEGG_STEROID_BIOSYNTHESIS
REACTOME_TRIGLYCERIDE_BIOSYNTHESIS
KEGG_TERPENOID_BACKBONE_BIOSYNTHESIS
KEGG_SPHINGOLIPID_METABOLISM

Supplementary Table 4: List of all TaqMan probes (Applied Biosystems, Foster City, CA, USA) used in this study

Gene	probe ID
SREBP2	Hs01081784_m1
SCAP	Hs00378725_m1
DHCR24	Hs00207388_m1
INSIG1	Hs01650979_m1
LDLR	Hs01092524_m1
HMGCS1	Hs00940429_m1
HMGCR	Hs00168352_m1
IDI1	Hs01057440_m1
FDFT1	Hs00926054_m1
LSS	Hs00158906_m1
ACTB	Hs99999903_m1

β-actin gene *ACTB* served as the housekeeping gene.

Supplementary Table 5: Resistance development of dtx-resistant sublines over time

**Month after selection start**	**HCT116/dtxA**		**HCT116/dtxB**		**HCT116/dtxE**	
	**IC_{50}# (µM)**	**relative resistance (-fold)**	**IC_{50}#(µM)**	**relative resistance (-fold)**	**IC_{50}#(µM)**	**relative resistance (-fold)**
4	3.3	1.3	8.9	4.4	0.3	3.8
6	15	3.5	14.8	4.2	1.7	38.6
8	> 60	> 20	> 50	> 25	2.3	36.3
12	> 100	> 28.6	> 100	> 40	3.7	55.7

IC_{50} values were calculated from dose-response curves and relative resistance was calculated by dividing the IC_{50} value of the respective resistant cell line by the IC_{50} value of the parental cell line HCT116/wt.

Supplementary Table 6: Cross-resistance of the three resistant sublines to classical chemotherapeutic agents

agent	HCT116/wt		HCT116/dtxA		HCT116/dtxB		HCT116/dtxE	
	IC_{50} #							
	mean	± SD	mean	± SD	mean	± SD	mean	± SD
5-FU (μM)	52	21	36.4	6.1	38.9	7.1	34.7	1.9
Oxaliplatin (μM)	0.8	0.1	0.5	0.0	1.0	0.2	0.4	0.1
Cisplatin (μM)	4.0	0.7	1.9*	0.8	2.2	0.2	4.4	0.4
Gemcitabin (μM)	0.4	0.2	0.4	0.2	0.4	0.2	0.3	0.0
Topotecan (nM)	27.4	9.3	26	11	8.2	3.6	25	19
Triapin (μM)	0.8	0.1	0.6	0.1	0.8	0.1	0.8	0.2

IC_{50} values were calculated from dose-response curves and are given as means ± SD from at least three independent experiments performed in triplicates. *$p < 0.05$.

Supplementary Table 7: Cross-resistance of the three resistant sublines to other fungal metabolites

Metabolites (μM)	HCT116/wt		HCT116/dtxA		HCT116/dtxB		HCT116/dtxE	
	IC_{50} #							
	mean	± SD	mean	± SD	mean	± SD	mean	± SD
Beauvericin	2.8	0.7	1.9	0.0	1.90	0.03	2.0	0.1
Enniatin B	2.6	0.3	2.5	0.2	2.5	0.2	2.2	0.1
Bassianolide	5.3	1.7	4.5	2.1	4.6	1.8	5.2	1.6

IC_{50} values were calculated from dose-response curves and are given as means ± SD from at least three independent experiments performed in triplicates.

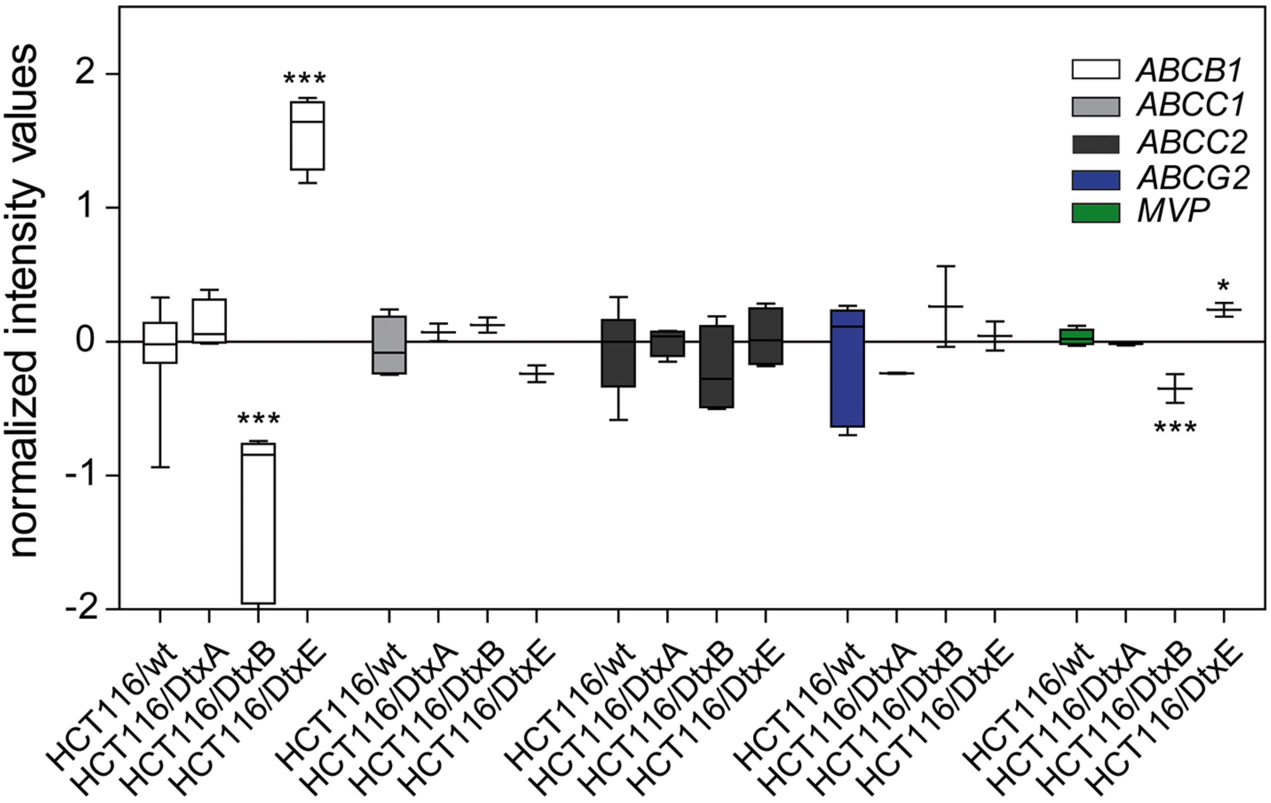

Supplementary Figure 1: mRNA expression of selected chemotherapy resistance genes. Normalized intensity values derived from gene expression array analysis of the indicated genes are given as Box plots for HCT116/wt cells and the dtxA-, dtxB- and dtxE-resistant sublines. $^{*}p<0.05$; $^{***}p<0.001$.

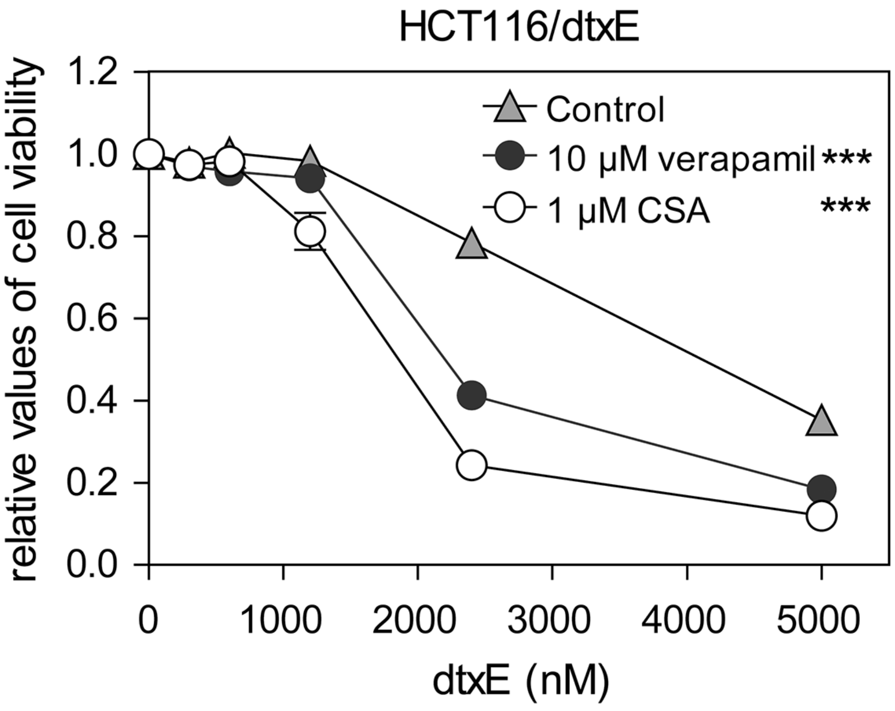

Supplementary Figure 2: Effects of ABCB1 inhibition on cell viability. Viability of HCT116/dtxE cells treated with increasing concentrations of dtxE alone (control) or in combination with 10 µM verapamil or 1 µM cyclosporin A as indicated is shown as mean ±SD from one representative experiment performed in triplicate. ***p<0.001.

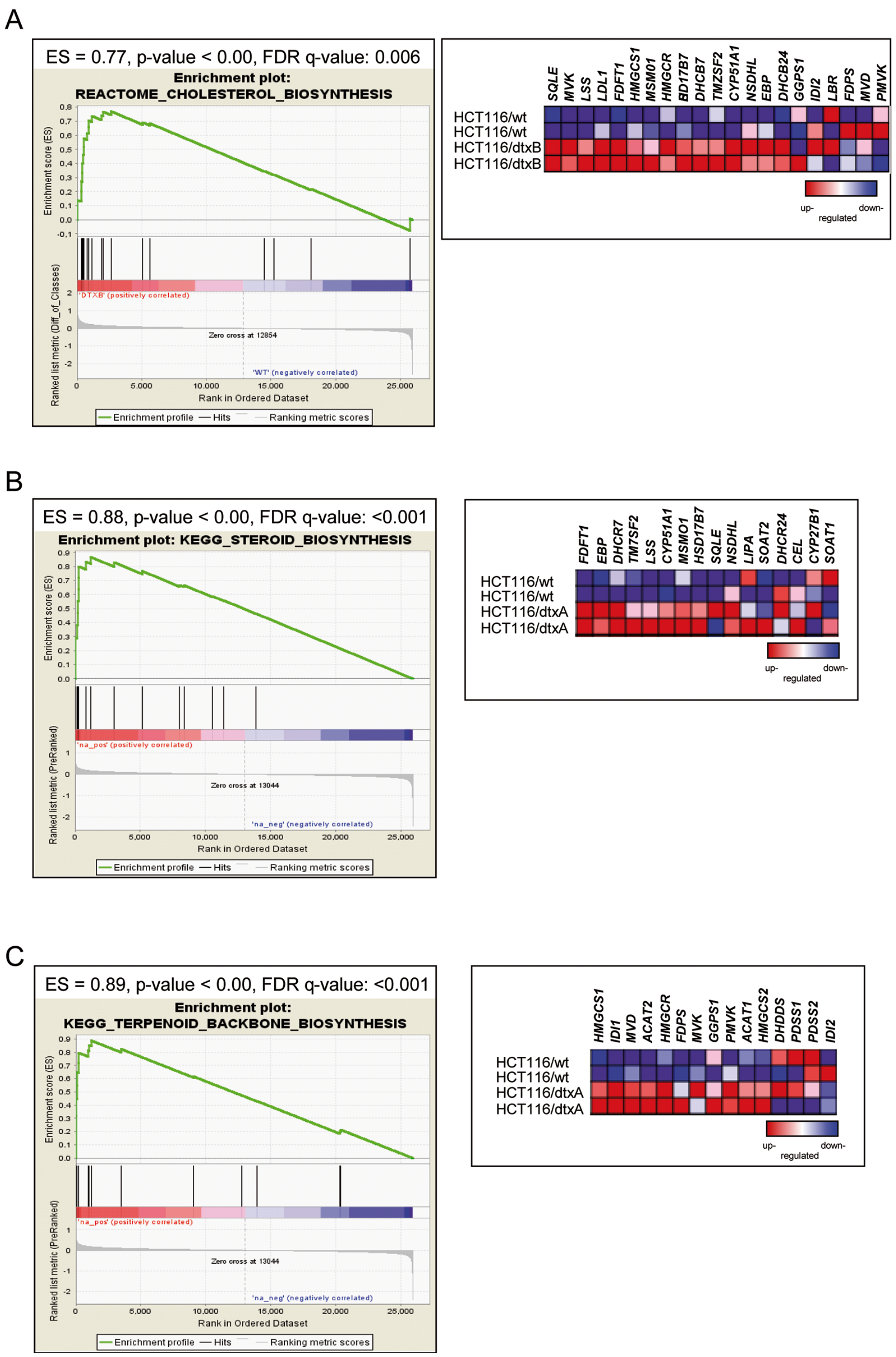

Supplementary Figure 3: GSEA and heat map of cholesterol associated gene sets. GSEA was performed with gene expression array data sets from either **(A)** HCT116/dtxB or (B, C) HCT116/dtxA as compared to the parental HCT116/wt cells. Highest enrichment scores were found for a multitude of gene sets related to the mevalonate pathway. Representatively, the gene sets (A) "Cholesterol_ Biosynthesis" of the Reactome database and **(B)** "Steroid_Biosynthesis" and **(C)** "Terpenoid_Backbone_Biosynthesis" of the KEGG database are shown (left) opposed to the respective heat map analyses (right).

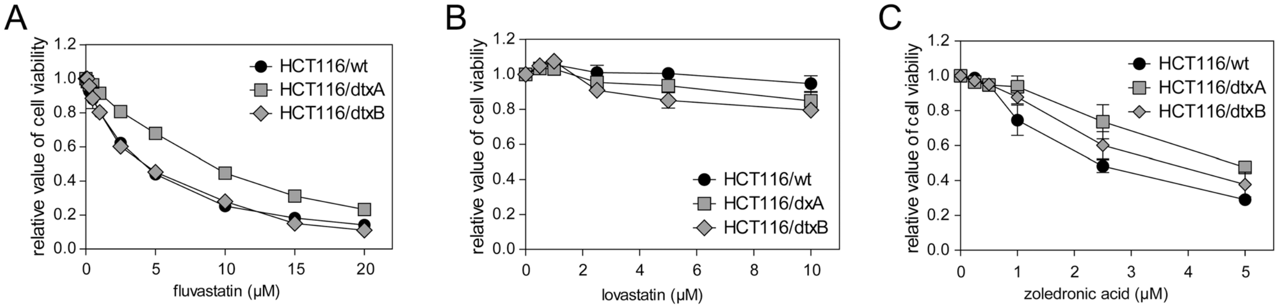

Supplementary Figure 4: Viability of dtx-sensitive HCT116/wt and the dtxA- and dtxB-resistant sublines treated with increasing concentrations of (A) fluvastatin, (B) lovostatin and (C) zoledronic acid for 72 h as indicated is shown as mean ±SD from one representative experiment performed in triplicate.

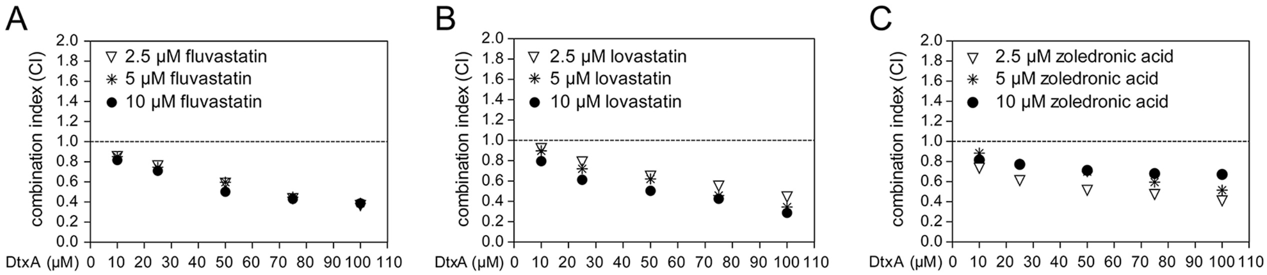

Supplementary Figure 5: Combination indices of dtxA and inhibitors of cholesterol synthesis. Combination indices (CI) based on cell viability data from HCT116/dtxA cells treated with dtxA in combination with increasing concentrations of **(A)** fluvastatin, **(B)** lovastatin or **(C)** zoledronic acid for 72 h are shown. CI < 1, synergism; CI = 1, additive effects; or CI > 1 antagonism.

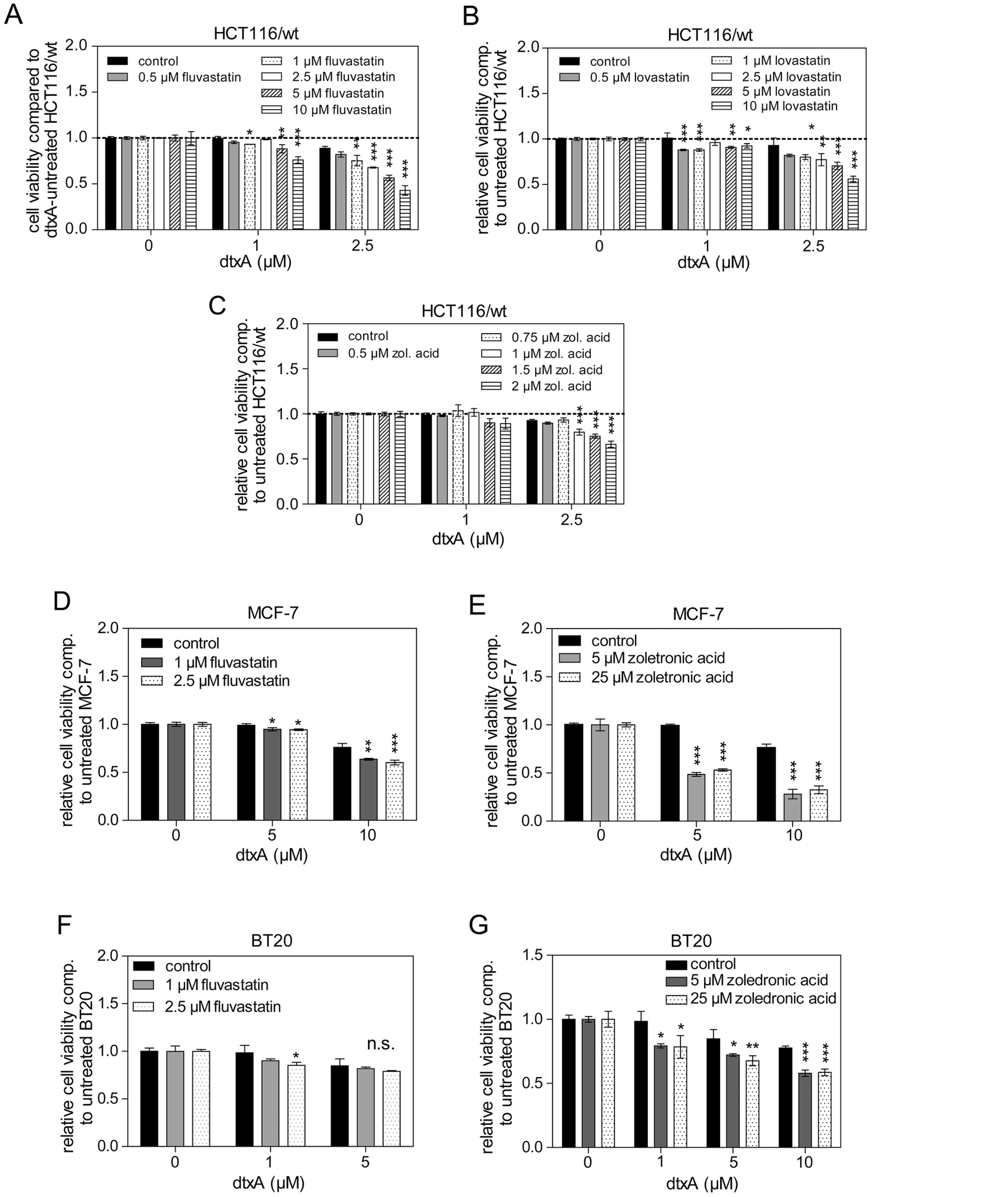

Supplementary Figure 6: Cytotoxicity of dtxA is enhanced in combination with different concentrations of mevalonate pathway inhibitors **(A, D, F)** fluvastatin, **(B)** lovastatin and **(C, E, G)** zoledronic acid in parental (A-C) HCT116/wt, (D, E) MCF-7 and (F, G) BT20 cells, latter two exerting intrinsically low sensitivity to destruxins, after 72h of treatment. Mean (± SD) cell viability normalized to the respective controls, are shown from one representative experiment performed in triplicates. $^{*}p < 0.05$, $^{**} < 0.01$, $^{***} < 0.001$;

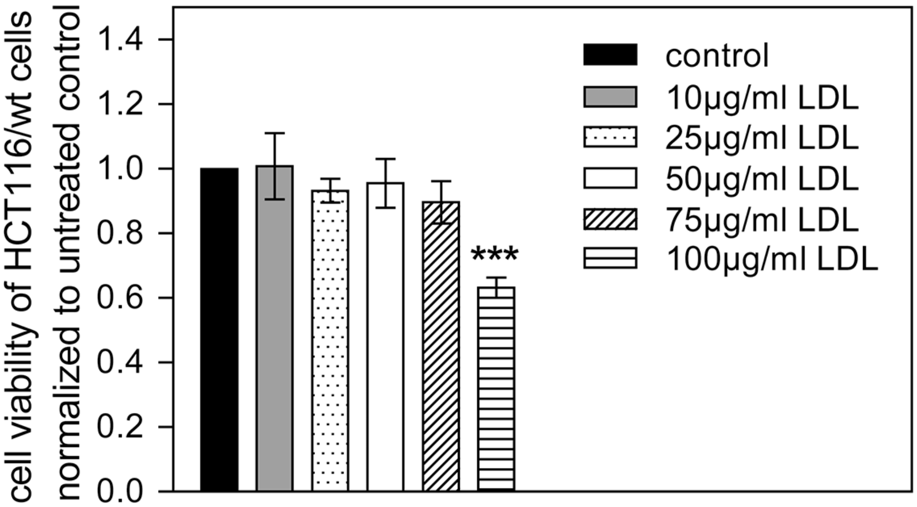

Supplementary Figure 7: Parental HCT116/wt cells were left untreated (control) or treated with increasing concentrations of LDL as indicated for 72 h in growth medium containing 1% FBS. Cell viability values are given relatively to the control and are shown as mean (± SD) of three independent experiments performed in triplicate. ***< 0.001;
